# The Nitric Oxide-Cyclic GMP Pathway Regulates FoxO and Alters Dopaminergic Neuron Survival in *Drosophila*

**DOI:** 10.1371/journal.pone.0030958

**Published:** 2012-02-29

**Authors:** Tomoko Kanao, Tomoyo Sawada, Shireen-Anne Davies, Hiroshi Ichinose, Kazuko Hasegawa, Ryosuke Takahashi, Nobutaka Hattori, Yuzuru Imai

**Affiliations:** 1 Research Institute for Diseases of Old Age, Juntendo University Graduate School of Medicine, Tokyo, Japan; 2 Department of Neurology, Juntendo University Graduate School of Medicine, Tokyo, Japan; 3 Department of Neuroscience for Neurodegenerative Disorders, Juntendo University Graduate School of Medicine, Tokyo, Japan; 4 Department of Neurology, Kyoto University Graduate School of Medicine, Kyoto, Japan; 5 CREST (Core Research for Evolutionary Science and Technology), JST, Saitama, Japan; 6 Institute of Molecular, Cell and Systems Biology, College of Medical, Veterinary and Life Sciences, University of Glasgow, Glasgow, Scotland, United Kingdom; 7 Department of Life Science, Graduate School of Bioscience and Biotechnology, Tokyo Institute of Technology, Yokohama, Japan; 8 Department of Neurology, National Hospital Organization, Sagamihara National Hospital, Sagamihara, Japan; Hertie Institute for Clinical Brain Research and German Center for Neurodegenerative Diseases, Germany

## Abstract

Activation of the forkhead box transcription factor FoxO is suggested to be involved in dopaminergic (DA) neurodegeneration in a *Drosophila* model of Parkinson's disease (PD), in which a PD gene product LRRK2 activates FoxO through phosphorylation. In the current study that combines *Drosophila* genetics and biochemical analysis, we show that cyclic guanosine monophosphate (cGMP)-dependent kinase II (cGKII) also phosphorylates FoxO at the same residue as LRRK2, and *Drosophila* orthologues of cGKII and LRRK2, DG2/For and dLRRK, respectively, enhance the neurotoxic activity of FoxO in an additive manner. Biochemical assays using mammalian cGKII and FoxO1 reveal that cGKII enhances the transcriptional activity of FoxO1 through phosphorylation of the FoxO1 S319 site in the same manner as LRRK2. A *Drosophila* FoxO mutant resistant to phosphorylation by DG2 and dLRRK (dFoxO S259A corresponding to human FoxO1 S319A) suppressed the neurotoxicity and improved motor dysfunction caused by co-expression of FoxO and DG2. Nitric oxide synthase (NOS) and soluble guanylyl cyclase (sGC) also increased FoxO's activity, whereas the administration of a NOS inhibitor L-NAME suppressed the loss of DA neurons in aged flies co-expressing FoxO and DG2. These results strongly suggest that the NO-FoxO axis contributes to DA neurodegeneration in *LRRK2-*linked PD.

## Introduction

PD, one of the most common movement disorders, is characterized by age-dependent impairments of several nervous systems including the midbrain DA system. The degeneration of DA neurons in the substantia nigra produces the prominent motor symptoms of PD. Postmortem inspections and studies with neurotoxin-based PD models suggest a multifactorial etiology involving inflammation, mitochondrial dysfunction, iron accumulation and oxidative stress. NO, a free gaseous signaling molecule, has also been implicated in PD [1], [2]. The signaling function of NO is dependent on the dynamic regulation of its synthase, NOS. There are three types of NOS, neuronal NOS (nNOS), endothelial NOS (eNOS) and inducible NOS (iNOS), in humans whereas the *Drosophila* genome has only a single orthologue, dNOS. High levels of nNOS and iNOS have been reported in the substantia nigra of PD patients [3], [4] and animal models of PD [5], [6]. Overproduction of NO is suggested to cause DNA damage, protein modifications and cell toxicity mainly mediated by the reactive species peroxynitrite, which may be generated with dopamine metabolism in DA neurons. In the etiology of PD, overproduction of NO could be caused either by upregulation of iNOS in activated glia cells [3], [5] or by an increase in intracellular calcium, for example, after glutamate excitotoxicity [7].

The discovery of genes linked to rare familial forms of PD has provided vital clues to understanding the cellular and molecular pathogenesis of the disease. Missense mutations in the *Leucine-rich repeat kinase 2 (LRRK2)/Dardarin* gene cause autosomal dominant late onset familial PD as well as sporadic PD [8], [9], [10]. The clinical symptoms and pathology caused by *LRRK2* mutations closely resemble those of the sporadic form of PD, suggesting that the LRRK2 pathogenic pathway may underlie the general PD etiology. The *LRRK2* gene encodes a large protein with multiple domains including a GTPase domain and a kinase domain [8], [9]. Several amino acid substitutions are identified as pathogenic mutations linked to PD [11]. Mutations in the kinase domain of human LRRK2 such as G2019S and I2020T have been reported to produce enhanced kinase activity *in vitro,* suggesting that gain-of-function mutations of LRRK2 cause neurodegeneration [12], [13], [14]. However, how these mutations present in the LRRK2 gene lead to the progressive loss of DA neurons and other associated pathologies is still unknown.

Because various key signaling pathways are conserved between humans and *Drosophila*, genetic and functional studies using *Drosophila* models for familial PD have revealed crucial signal transductions that affect the pathogenesis of PD [15]. We have previously reported that a *Drosophila* LRRK2 orthologue, dLRRK phosphorylates *Drosophila* FoxO (dFoxO) at Ser259, which stimulates the expression of a pro-apoptotic dFoxO target, *hid,* and leads to neurodegeneration in *Drosophila* [16]. The event was further enhanced by transgenic expression of pathogenic dLRRK proteins such as dLRRK I1915T (corresponding to I2020T in humans). However, a kinase-dead form of dLRRK (dLRRK 3KD) did not completely suppress a synergic effect caused by the co-expression of dFoxO with dLRRK, suggesting that some other factor(s) modulates this pathway. Here, we report that cGKII also phosphorylates FoxO and activates FoxO-transcriptional activity in the same manner as LRRK2/dLRRK by using biochemical studies of mammalian cGKII and FoxO1. Moreover, by using *Drosophila* models, our data suggest that NO signaling and its downstream effector cGKII/DG2 contribute to DA neurodegeneration.

## Results

### cGK genetically interacts with FoxO and activates FoxO activity

**Figure 1:**
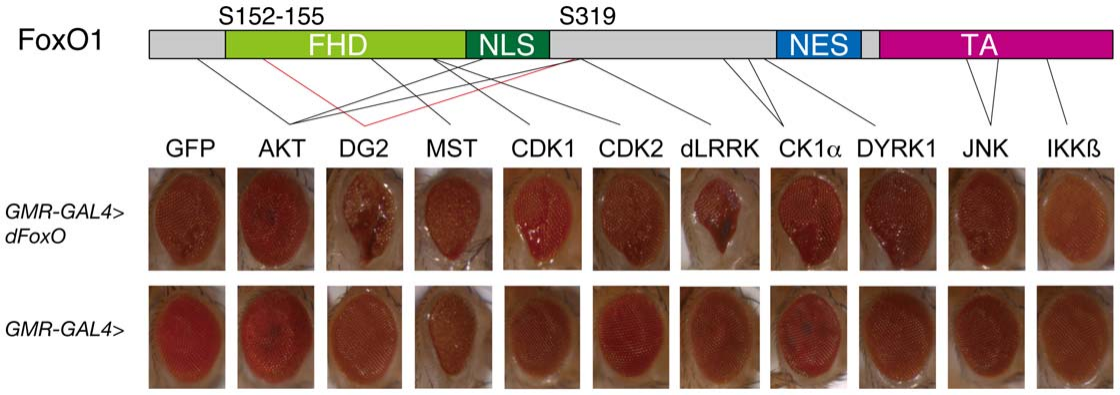
Screening of kinases that affect the eye phenotypes caused by dFoxO. *Drosophila* orthologues of reported FoxO kinases were expressed with (upper row) or without (lower) dFoxO in *Drosophila* eyes using the *GMR-GAL4* driver. GFP served as a control. The *Drosophila* DG2 is presumably functionally equivalent to the vertebrate cGKII. Reported phosphorylation sites and newly identified sites that are phosphorylated by cGKII (S152–155 and S319) in human FoxO1 are indicated by black and red lines, respectively. FHD, forkhead domain; NLS, nuclear localization signal; NES, nuclear export signal; TA, transactivation domain. Overexpressing lines used for crosses are: *UAS-GFP* (GFP), *UAS-AKT1* (AKT), *UAS-DG2* (DG2), *UAS-hippo* (MST), *UAS-CDK1-Myc* (CDK1), *UAS-CDK2-Myc* (CDK2), *UAS-dLRRK* (dLRRK), *CkIα^EP1555^* (CK1α), *mnb^EY14320^* (DYRK1), *UAS-bsk* (JNK), *UAS-dIKKß* (IKKβ).

**Figure 2:**
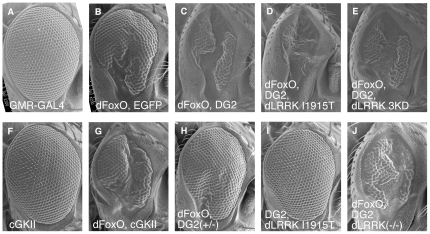
DG2 as well as dLRRK additively enhances FoxO-mediated developmental defects in the *Drosophila* eye. (**A–J**) SEM images of the eye of flies expressing the indicated genes. The genotypes are: *GMR-Gal4* (**A**), *GMR-Gal4/UAS-EGFP* (**B**), *UAS-DG2; GMR-Gal4, UAS-dFoxO* (**C**), *UAS-DG2; GMR-Gal4, UAS-dFoxO; UAS-dLRRK I1915T* (**D**), *UAS-DG2; GMR-Gal4, UAS-dFoxO; UAS-dLRRK 3KD* (**E**), *GMR-Gal4; UAS-cGKII* (**F**), *GMR-Gal4, UAS-dFoxO; UAS-cGKII* (**G**), *GMR-Gal4, UAS-dFoxO/DG2^k04703^* (**H**), *UAS-DG2; GMR-Gal4; UAS-dLRRK I1915T* (**I**), *UAS-DG2; GMR-Gal4, UAS-dFoxO; e03680/e03680* (**J**).

We previously reported a genetic interaction between FoxO and LRRK2/dLRRK in *Drosophila* [16]. To identify components of the LRRK2-FoxO signaling pathway, we screened for modifiers (Fig. 1 and Fig. S1A). Kinases reported to affect the activity of FoxO were expressed with dFoxO in the *Drosophila* eye. As reported, transgenic expression of AKT suppressed FoxO-mediated developmental defects in the eye. The expression of MST/Hippo resulted in extensive degeneration, which did not appear to be dependent on FoxO (Fig. 1). Expression of one of the *Drosophila* cGMP-dependent kinases (cGKs), DG2, leads to strong optic degeneration in conjunction with dFoxO (Fig. 1 and ), while the other kinases had little effect on the developmental defects caused by FoxO (Fig. 1). Removal of one copy of the *dg2* gene improved the defects, suggesting that endogenous DG2 activity contribute to the dFoxO-mediated neurodegeneration (Fig. 2H compared with B).

Next we examined whether DG2 is an upstream kinase of dLRRK, or whether DG2 acts independently of dLRRK by means of a combination of genetic interaction tests, reporter assays for FoxO and *in vitro* kinase assay. Co-expression of dLRRK harboring a PD-related mutant I1915T together with DG2 dramatically enhanced the toxicity of dFoxO (Fig. 2D compared with C). However, expression of dLRRK 3KD or removal of the dLRRK gene did not suppress the eye phenotype caused by dFoxO-DG2 at all (Fig. 2E and J compared with C). Co-expression of DG2 and dLRRK I1915T produced a normal eye, suggesting that the phenotype is dependent on the level of dFoxO protein (Fig. 2I compared with D).

**Figure 3:**
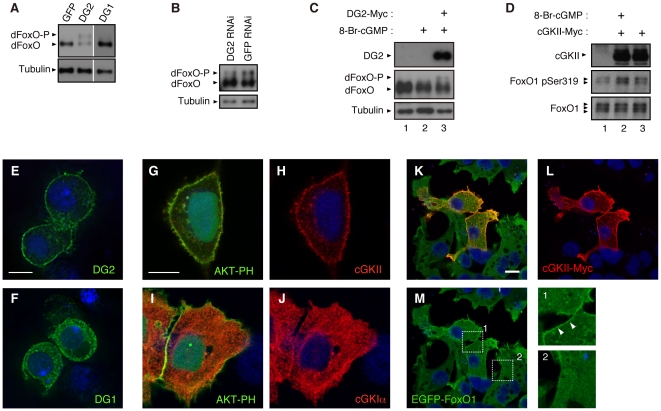
DG2 modulates FoxO *in vivo*. (**A**) dFoxO and the indicated transgenes were expressed in the *Drosophila* eyes using the *GMR-GAL4* driver. Extracts from brain tissues were subjected to western blot analysis. dFoxO-P; a phosphorylated form of dFoxO. (**B**) DG2 RNAi or GFP RNAi constructs were expressed in the *Drosophila* brain using the *elav-GAL4* driver. Western blot analysis for endogenous dFoxO was carried out as in (**A**). (**C**) *Drosophila* S2 cells were transfected with or without C-terminally Myc-tagged DG2 (DG2-Myc). Thirty-six hrs post transfection, cells were treated with or without 10 µM 8-Br-cGMP for 30 min. Cell lysate were then subjected to western blot analysis. (**D**) Human 293T cells were transfected with or without cGKII-Myc, and were treated with 8-Br-cGMP as in (**C**). Phosphorylation of the S319 site in endogenous FoxO1 was detected with phospho-specific antibody. (**E, F**) S2 cells expressing DG2-Myc (**E**) or DG1-Myc (**F**) were visualized with anti-Myc antibody (green), by counterstaining with DAPI (blue color). (**G–J**) HeLa cells expressing AKT-PH-GFP (green) along with cGKII-Myc (**G, H**) or cGKI-Myc (**I, J**) were visualized with anti-Myc antibody (red), by counterstaining with DAPI (blue color). AKT-PH-GFP was used for a marker protein of the plasma membrane [62]. (**K**–**M**) Flp-In T-REx-293 cells harboring EGFP-FoxO1 gene were transiently transfected with cGKII-Myc, and EGFP-FoxO1 was induced with doxycycline. Enlarged views of the plasma membrane regions in cGKII-positive (Box1) and negative (Box2) cells are also shown in (**M**). Accumulation of FoxO1 along with cGKII in the plasma membrane is indicated by arrowheads. Scale bars = 5 µm for (**E, F**), 25 µm for (**G–J**) and 10 µm for (**K–M**).

Co-expression of dFoxO with DG2, but not GFP or DG1, in *Drosophila* eyes caused appearance of a slower migrated dFoxO protein in western blot analysis (Fig. 3A), which indicates phosphorylation of dFoxO [16]. Consistent with the result, knockdown of DG2 decreased a phosphorylated form of endogenous dFoxO in *Drosophila* brain tissue (Fig. 3B). In *Drosophila* S2 cells, transient expression of DG2 together with 8-bromoguanosine-3′, 5′-cyclic monophosphate (8-Br-cGMP), a membrane permeable analogue for cGMP, also stimulates phosphorylation of endogenous dFoxO (Fig. 3C, lane 3).

**Figure 4:**
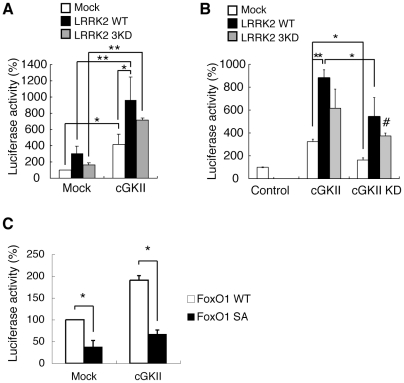
cGKII stimulates FoxO-transcriptional activity. (**A, B**) cGKII and LRRK2 additively stimulate FoxO-transcriptional activity. FoxO-transcriptional activity was measured in extracts prepared from 293T cells transfected with the indicated plasmids and a plasmid for FoxO1, a FoxO reporter plasmid containing *Firefly* luciferase, and a plasmid for *Renilla* luciferase to monitor the transfection efficiency. The relative FoxO-transcriptional activity (*Firefly* luciferase activity) normalized to *Renilla* luciferase activity is presented. Data are presented as the mean ± SE for three independent experiments. β-galactosidase (Mock) served as a transfection control. (**C**) Introduction of the S319A (SA) mutation in FoxO1 reduced FoxO activity. Data are presented as the mean ± SE for three independent experiments. *, *p*<0.05; **, *p*<0.01. Co-transfection of kinase-dead forms of cGKII and LRRK2 also sitmulated FoxO (#, *p*<0.05 *vs.* Control in **B**).

Two groups of cGKs, the soluble type I (cGKI α and β) and the membrane-bound type II (cGKII), have been reported in vertebrates. In *Drosophila*, there are two genes encoding cGK, namely *dg1* and *dg2* [17]. As reported [18], the gene products DG1 and DG2 are located in the cytoplasm and at the cytoplasmic membrane, respectively (Fig. 3E and F). Interestingly, expression of DG1 had little effect on the degeneration of the eye mediated by dFoxO, suggesting that DG1 and DG2 have different roles *in vivo* (Fig. S1B, S2B and S2C). Although predictions of amino acid sequence indicate that DG2 is more similar as a cGKI α/β homologue [19], their subcellular distribution suggests that DG2 is functionally more similar to cGKII (Fig. 3G–J) [18], [20], [21]. Consistent with the idea, transgenic expression of human cGKII exacerbated eye degeneration by dFoxO (Fig. 2G compared with B) whereas expression of cGKII alone did not affect the eye development (Fig. 2F). Interestingly, cGKII appeared to recruit FoxO1 to the cytoplasmic membrane of human cultured cells (Fig. 3K–M) while there was no evidence that cGKI associates with cGKII *in vivo* (Fig. S3). In addition, we observed that cGKII is abundantly expressed in DA neurons in the substantia nigra of mice (Fig. S4). We then focused on mammalian cGKII as a cGK that might be associated with the pathology of PD. Reporter assays for FoxO transcriptional activity revealed that cGKII stimulated FoxO activity in cultured mammalian cells and that co-expression of hLRRK2 with cGKII caused a 3-fold increase in FoxO activity (Fig. 4A). A kinase-dead form of hLRRK2 (hLRRK2 3KD) did not suppress the activation of FoxO by cGKII to the control level. Similarly, a kinase-dead form of cGKII (cGKII KD) failed to suppress FoxO's activation by LRRK2 (Fig. 4B). The results of the genetic interaction tests and the reporter assays suggested that cGKII and LRRK2 have additive effects on the regulation of FoxO activity.

### cGK directly phosphorylates FoxO in vitro

**Figure 5:**
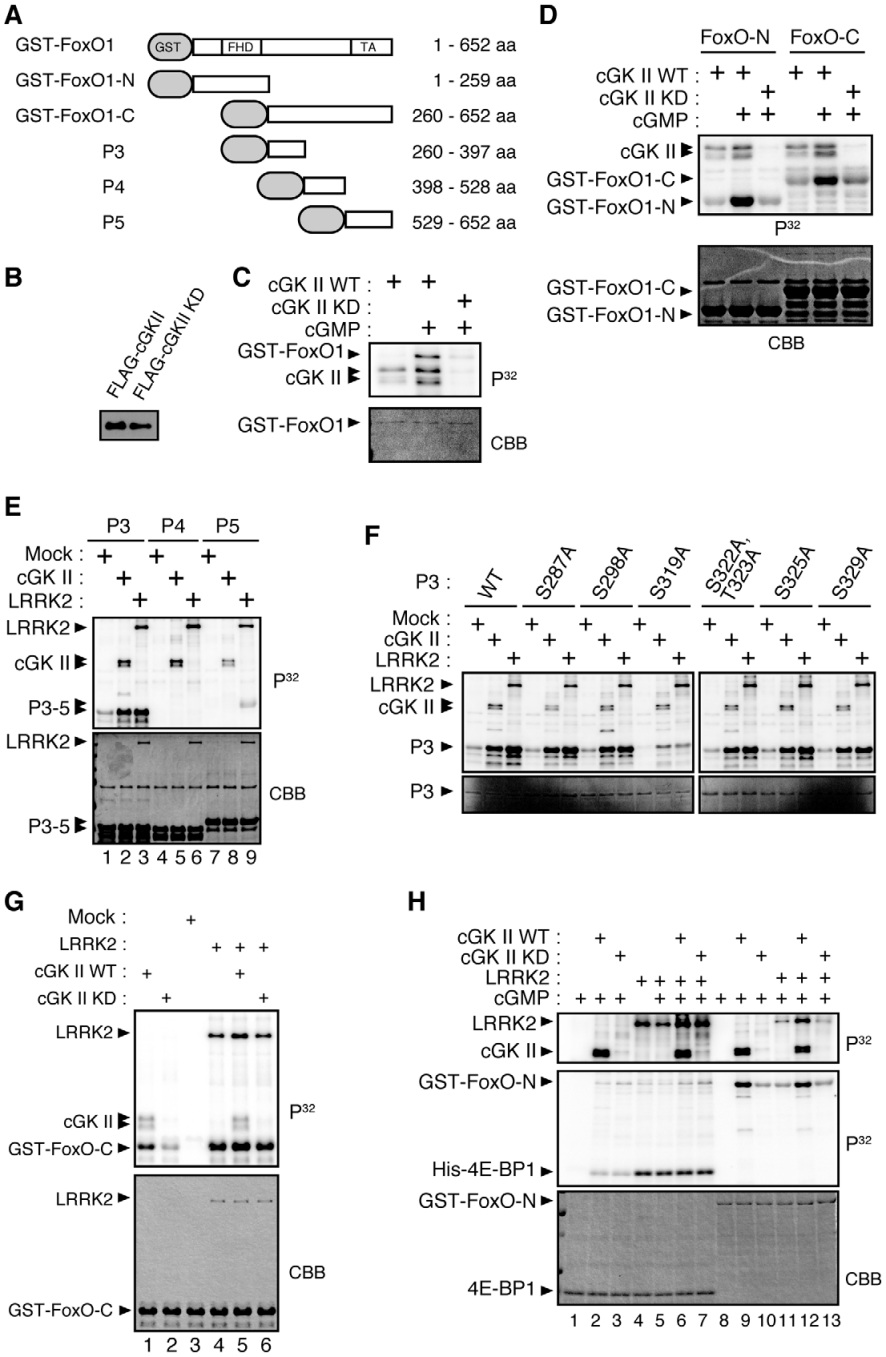
cGKII phosphorylates FoxO1 *in vitro*. (**A**) The recombinant FoxO1 proteins used as substrates. GST, GST-tag. Numbers indicate corresponding amino acid residues of FoxO1. (**B**) FLAG-tagged cGKII and FLAG-cGKII KD were immunoprecipitated from FLAG-tagged cGKII or FLAG-cGKII KD-transfected 293T cells as kinase sources. Western blotting confirmed that the amounts of the two proteins obtained were similar. (**C, D**) *In vitro* kinase assays of cGKII using recombinant GST-FoxO1 as a substrate. In the presence of cGMP, cGKII WT but not cGKII KD phosphorylated GST-FoxO, GST-FoxO-N, and GST-FoxO-C. Autoradiography (P^32^) and Coomassie brilliant blue (CBB) staining of the gels are shown. Note cGKII proteins by CBB staining were difficult to detect in spite of the presence of autophosphorylation signals of cGKII (cGKII in P^32^) (**E**) cGKII and LRRK2 phosphorylated the P3 but not P4 or P5 protein. Autophosphorylation signals of cGKII and LRRK2 are also shown (cGKII and LRRK2 in P^32^). The mock immunoprecipitate (Mock) served as a control. (**F**) *In vitro* kinase assay using P3 and a series of P3 mutants where the candidate phosphorylation residues are replaced with alanine (refer to [16] for information on the mutated residues). The phosphorylation by cGKII or LRRK2 was decreased in the P3 S319A mutant. (**G**) Co-incubation of cGKII and LRRK2 enhanced GST-FoxO-C phosphorylation. (**H**) cGKII failed to stimulate LRRK2 kinase activity and LRRK2 failed to stimulate cGKII kinase activity. His-tagged 4E-BP1 and GST-FoxO-N served as LRRK2-specific and cGKII-specific substrates, respectively.

Previously, we have demonstrated that LRRK2 phosphorylates, and enhances the neurotoxic activity of, FoxO. Using *in vitro* kinase assays, we tested whether cGKII stimulates the kinase activity of LRRK2 through phosphorylation, or whether cGKII directly activates FoxO as shown in a study on LRRK2 [16]. We transfected HEK293 cells with a FLAG-tagged cGKII or FLAG-cGKII KD plasmid and affinity-purified these proteins using anti-FLAG columns (Fig. 5B). We observed that cGKII WT but not KD specifically phosphorylated GST-FoxO1 in the presence of cGMP (Fig. 5C), and that cGKII targeted at least two sites of FoxO1, which were in FoxO-N and FoxO-C (Fig. 5A and D). A previous report has shown that cGKIα phosphorylates the human FoxO1 forkhead domain mainly at S152–155 and S184, by which the DNA-binding activity of FoxO1 is abolished [22]. We found that cGKII also phosphorylates FoxO1 at S152–155 and that these residues are major sites of phosphorylation in FoxO-N (Fig. S5A and B). However, the replacement of serine with alanine at S152–155 had little effect on the FoxO-transcriptional stimulation by cGKII and the binding to 14-3-3ε protein, which regulates the cytosolic localization of FoxO, in this context (Fig. S5C and D). Next, we determined phosphorylation sites in FoxO-C. Experiments with several truncated FoxO1 mutants narrowed down the phosphorylation sites in FoxO-C and identified S319 as a major phospho-residue targeted by cGKII (Fig. 5E and F). We also confirmed that overexpression of cGKII in the presence of 8-Br-cGMP stimulates the phosphorylation of the FoxO1 S319 site in human cultured cells (Fig. 3D, lane 2). Although cGKIα also phosphorylated GST-tagged full-length FoxO1 *in vitro*, the S319 site did not appear to be a major phosphorylation site (Fig. S6). The S319 site was also targeted by LRRK2 as shown previously (Fig. 5F) and co-incubation of cGKII and LRRK2 enhanced phosphorylation of the FoxO-C fragment in *in vitro* kinase assays (lane 5 compared with lane 1 in Fig. 5G). In contrast to the phosphorylation of FoxO at S152–155, the replacement of serine with alanine at S319 suppressed FoxO-transcriptional activity and abolished cGKII-mediated stimulation of FoxO, suggesting that phosphorylation at S319 has a major effect on the activity mediated by cGKII as well as LRRK2 (Fig. 4C) [16].

### cGK phosphorylates LRRK2, but does not affect the kinase activity of LRRK2 in vitro

To examine the possibility that cGKII activates the kinase activity of LRRK2, or that LRRK2 activated cGKII, we further performed *in vitro* kinase assays using 4E-BP1 and FoxO-N as substrates (Fig. 5H). As reported [14], LRRK2 specifically phosphorylated 4E-BP1, which is not dependent on cGMP, while cGKII failed to do so (lanes 4 and 5 compared with lane 2 in Fig. 5H). cGKII and cGKII KD had little effect on the kinase activity of LRRK2 toward 4E-BP1 (lanes 6 and 7 *vs*. lanes 4 and 5 in Fig. 5H). cGKII but not cGKII KD or LRRK2 effectively phosphorylated FoxO-N (lane 9 compared with lanes 10 and 11 in Fig. 5H). Again LRRK2 had little effect on the kinase activity of cGKII toward FoxO-N (lane 12 compared with lanes 9 and 13 in Fig. 5H). However, cGKII also appeared to phosphorylate LRRK2 without modifying the kinase activity of LRRK2 (lane 6 *vs.* lanes 5, and lane 12 *vs.* lane 11 in Fig. 5H and Fig. S7). The *in vitro* observation that cGKII and LRRK2 act independently was consistent with the results of the genetic test (Fig. 2) and the reporter assay (Fig. 4).

### Phosphorylation of FoxO by DG2 as well as dLRRK causes DA neurodegeneration

**Figure 6:**
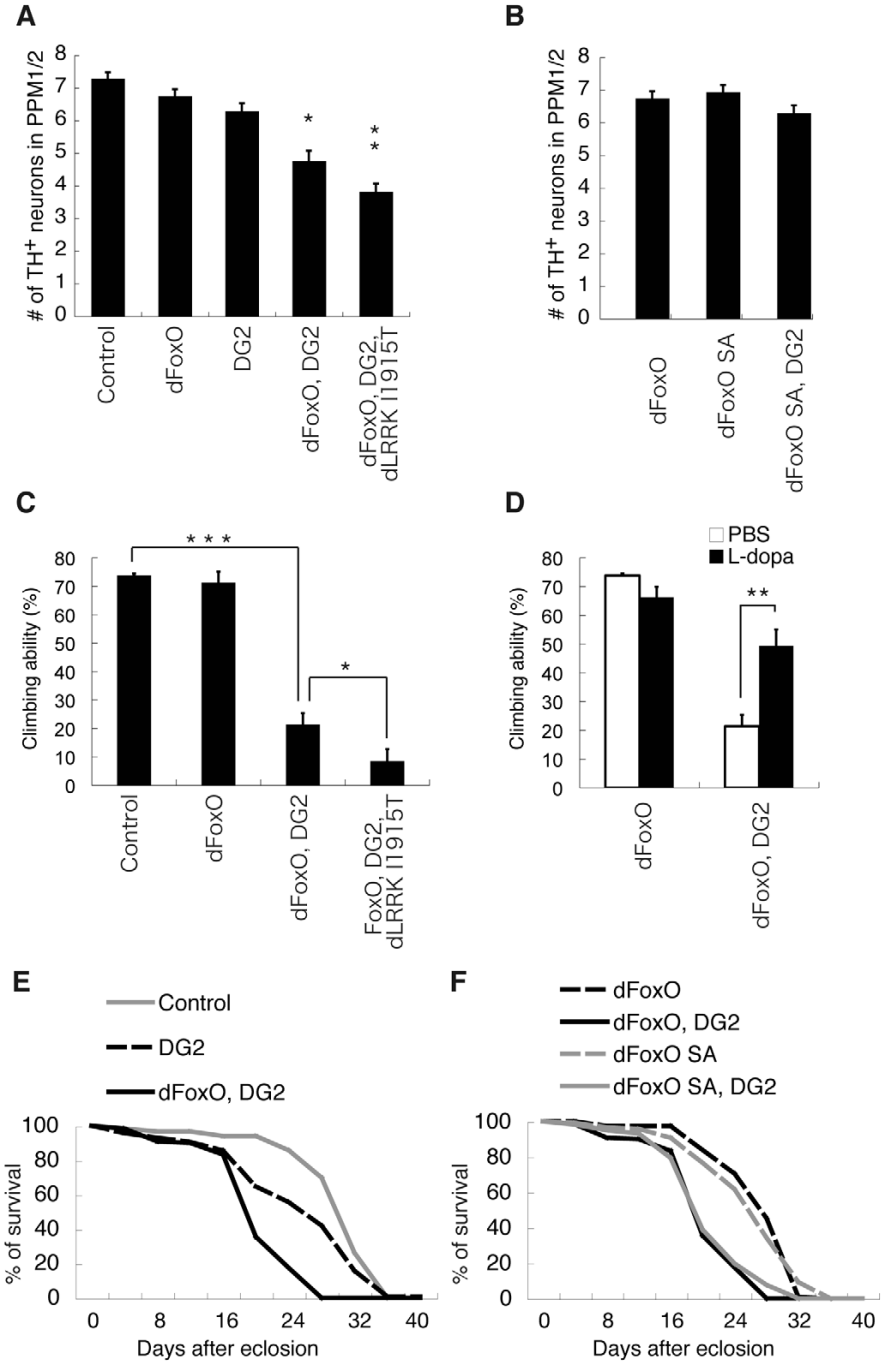
Neuronal activation of dFoxO by DG2 affects the maintenance of DA neurons in *Drosophila*. (**A**) The number of protocerebral posterior medial (PPM) 1/2 clusters of Tyrosine hydroxylase (TH)-positive DA neurons in 24-day-old adult flies. Neuron-specific expression of dFoxO, dLRRK I1915T and/or DG2 was induced following the administration of the activator RU486 (25 µg/mL) in the *elav-GeneSwitch-GAL4* (*elav-GS*) crosses. *elav-GS*/+ served as a control. Data are presented as the mean ± SE for three repeated experiments (*, *P*<0.05; **, *p*<0.01). (**B**) Co-expression of the dFoxO S259A (SA) mutant with DG2 suppressed the loss of PPM 1/2 TH-positive neurons. Flies were treated as in (**A**). (**C**) Adult aged flies expressing dFoxO and DG2 under the control of *elav-GS* showed motor defects, while the expression of dFoxO alone had little effect. The values represent means ± SE for 20 trials in six independent experiments (*, *p*<0.05; ***, *p*<0.001). (**D**) Treatment with 1 mM L-DOPA in phosphate-buffered saline (PBS), but not with PBS alone, for 4 days rescued the loss of climbing ability in dFoxO and DG2-expressing flies. dFoxO served as a control. The values represent means ± SE for 20 trials in six independent experiments (**, *p*<0.01). (**E**) Flies from each genotype were subjected to survival assays at 29°C. *elav-GS*/+ served as a control. Female adults (*n* = 119–121) were fed yeast paste containing 25 µg/mL_ RU486. Expression of DG2 shortened lifespan compared with the control (DG2 *vs.* Control, *p*<0.01; dFoxO, DG2 *vs.* Control, *p*<0.0001). (**F**) Flies from each genotype (*n* = 119–122) were subjected to survival assays as in (**E**). Pan-neuronal expression of dFoxO SA alone had no significant effect on lifespan when compared with that of dFoxO. Co-expression of dFoxO SA with DG2 failed to attenuate the effect of dFoxO-DG2 combination on lifespan (dFoxO SA, DG2 *vs.* dFoxO, DG2; *p* = 0.485).

**Figure 7:**
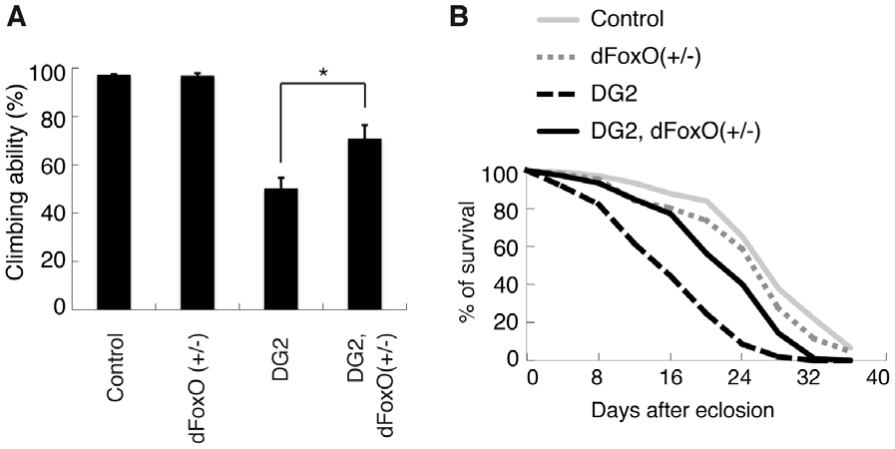
Reduction of endogenous dFoxO activity suppresses DG2-mediated toxicity. (**A**) The climbing activity was measured as in Figure 6C. The values represent means ± SE for 20 trials in three independent experiments (*, *p*<0.05). (**B**) Survival assays of female adults (*n* = 105–106) were performed as in Figure 6E. DG2 *vs.* DG2, dFoxO (+/−), *p*<0.01; DG2, dFoxO (+/−) *vs.* Control, *p*<0.01. The genotypes are: *elav-GS/+* (Control), *UAS-DG2*; *elav-GS* (DG2), *elav-GS/dFoxO^21^* (dFoxO (+/−)), *UAS-DG2*; *elav-GS/dFoxO^21^* (DG2, dFoxO (+/−)).

We next examined the pathological consequence of the phosphorylation of FoxO by DG2 and dLRRK in *Drosophila*. Ubiquitous or pan-neuronal expression of DG2 or dFoxO using GAL4 drivers for constitutive expression caused death. We then employed the mifepristone-inducible GAL4 system (GeneSwitch-GAL4) that drives the tissue-specific expression of upstream activating sequence (UAS)-constructs in post-mitotic cells. Pan-neuronal co-expression of dFoxO with DG2, but not the expression of either dFoxO or DG2 alone, caused significant neuronal loss in the PPM1/2 cluster Tyrosine hydroxylase (TH)-positive neurons of the adult brain (Fig. 6A). Expression of dLRRK I1915T exacerbated the neurotoxicity mediated by dFoxO and DG2 co-expression (Fig. 6A). In this context, the introduction of the S259A mutation, which corresponds to S319A in human FoxO1, attenuated the toxic interaction of dFoxO with DG2 (Fig. 6B). Consistent with the viability of TH-positive neurons, the motor activity of the flies expressing dFoxO and DG2 was impaired (Fig. 6C). Co-expression of dLRRK I1915T further worsened the motor dysfunction (Fig. 6C). Treatment with 1 mM L-3,4-dihydroxyphenylalanine (L-DOPA) significantly improved the locomotor activity of dFoxO and DG2-coexpressing flies (Fig. 6D), suggesting that the reduction in motor activity reflects DA degeneration. The expression of only DG2 mildly affected lifespan (Fig. 6E), whereas the co-expression of DG2 and dFoxO significantly shortened lifespan (Fig. 6E). However, the dFoxO S259A mutation failed to suppress the decrease in lifespan caused by the co-expression of dFoxO and DG2, suggesting that the toxic interaction of DG2 with dFoxO that affects lifespan is produced by a different mechanism rather than phosphorylation at S259 by DG2 (Fig. 6F). We then examined whether endogenous dFoxO contributes to DG2-mediated toxicity in *Drosophila* (Fig. 7A and B). Pan-neuronal expression of DG2 alone by the GeneSwitch-GAL4 driver caused mild motor defect (Fig. 7A). Removal of one copy of functional FoxO allele had little effect on the motor function (Fig. 7A) and lifespan (Fig. 7B) whereas it partly suppressed DG2-mediated motor dysfunction (Fig. 7A) and reduction in lifespan (Fig. 7B). These results suggested that endogenous dFoxO is also involved in neurodegeneration by DG2.

### NO signal leads to DA neurodegeneration through DG2-FoxO

**Figure 8:**
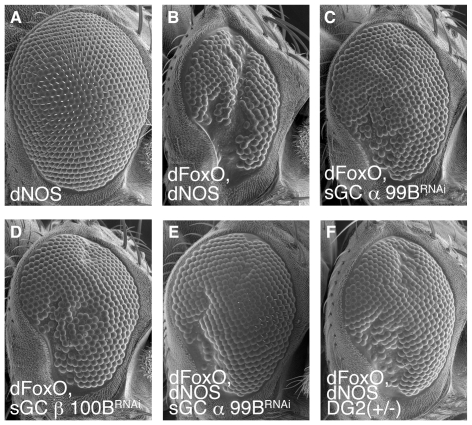
NO signal is involved in FoxO-mediated neurodegeneration. (**A–D**) SEM images of the eyes of flies expressing the indicated genes. The genotypes are: *GMR-Gal4/UAS-dNOS* (**A**), *GMR-Gal4, UAS-dFoxO/UAS-dNOS* (**B**), *GMR-Gal4, UAS-dFoxO; UAS-sGCα99B^RNAi^* (**C**), *GMR-Gal4, UAS-dFoxO; UAS-sGCβ100B^RNAi^* (**D**), *GMR-Gal4, UAS-dFoxO/UAS-dNOS; UAS-sGCα99B^RNAi^* (**E**), *GMR-Gal4, UAS-dFoxO/UAS-dNOS, DG2^k04703^* (**F**).

**Figure 9:**
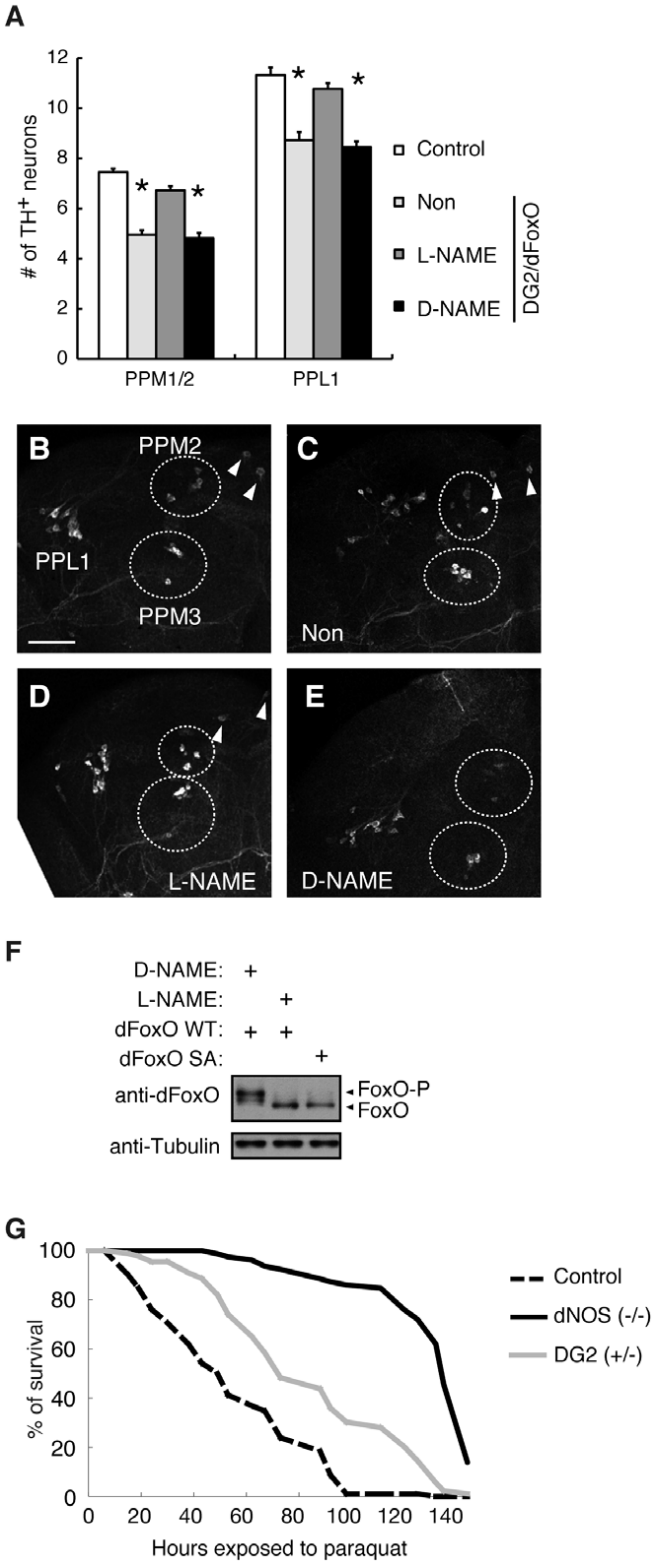
Inhibition of NO signal improves DG2-dFoxO-mediated DA neurodegeneration. (**A**) Newly eclosed normal *w-* flies (Control) or transgenics harboring *elav-GS>UAS-dFoxO/UAS-DG2* (*n* = 22 in each) were fed a yeast paste containing 50 µg/mL_ RU486 with or without 10 mM L-NAME or D-NAME every 4 days at 29°C. The graph presents the number of PPM 1/2 and PPL1 clusters of TH-positive neurons in 20-day-old adult flies. PPM1 and PPM2 clusters were counted together. Data are presented as the mean ± SE for three experiments (*, *p*<0.05 *vs.* Control). PPL, the protocerebral posterior lateral. Non; RU486 only. (**B**) A representative image of TH-positive PPL1, PPM1, PPM2 (upper circle) and PPM3 (lower circle) neurons of a wild-type *w-* adult fly. Arrowheads indicate a pair of PPM1 neurons. Bar = 50 µm. (**C**–**E**) Representative images of TH-positive neurons treated as in **A**. (**F**) Brain tissues of dFoxO transgenic flies treated with L-NAME or D-NAME were subjected to western blot analysis with anti-dFoxO. dFoxO SA mutant was also included as a non-phosphorylated control. Transgenes were expressed by the *elav-GS* driver. (**G**) Reduction of dNOS and DG2 activities confers stress resistance against 2 mM paraquat treatment. dNOS (−/−) *vs.* Control, *p*<0.0001; DG2 (+/−) *vs.* Control, *p*<0.01. The genotypes are: *w-* (Control), *dNOS^Δ15^/dNOS^Δ15^* (dNOS (−/−)), *DG2^k04703^*/+ (DG2 (+/−)).

The activation of cGK requires cGMP. cGMP is generated by the NO-mediated activation of sGCs as well as ligands-mediated activation of receptor GCs [23], [24], [25]. However, as NO generated by NOS has been implicated in PD, the role of NOS-sGC was investigated via functional assays in *Drosophila*. We tested whether the *Drosophila* NO signal components dNOS and sGC are indeed involved in FoxO and DG2-mediated DA neurodegeneration in *Drosophila* (Fig. 8). Genetic interaction tests showed that co-expression of dNOS enhances the FoxO-mediated degeneration in the eye (Fig. 8B). In contrast, knockdown of sGC α or β subunits partially improved the phenotype of dFoxO expression (Fig. 8C and D). Moreover, knockdown of sGCα or removal of one copy of the DG2 genes improved the eye degeneration caused by co-expression of dFoxO with dNOS (Fig. 8E and F compared with B). In the context of pan-neuronal expression of FoxO and DG2 in *Drosophila*, treatment with a NOS inhibitor, Nω-Nitro-L-Arginine-Methyl-Ester (L-NAME), but not the inactive D-enantiomer D-NAME, significantly suppressed loss of the PPM1/2 and PPL1 cluster DA neurons (Fig. 9A–E). In this setting, L-NAME treatment specifically reduced phosphorylation of dFoxO (Fig. 9F). The endogenous function of dNOS-DG2 signaling in DA neurodegeneration was estimated by survival assays of DG2 or dNOS mutant flies administrated with a PD-related toxin, paraquat, where both mutant lines showed significant resistances to paraquat (Fig. 9G). These results suggested that DG2/cGKII activated by NO signal could affect the survival of DA neurons through FoxO.

## Discussion

We have previously demonstrated that dLRRK/LRRK2 phosphorylates and stimulates FoxO, which confers neurotoxic activity to FoxO, activating the expression of pro-apoptotic proteins such as Bim/Hid [16]. Searching for LRRK2-FoxO signaling components, we found that *Drosophila* cGK DG2 also exacerbates FoxO-mediated neurotoxicity. The current study suggests that cGKII/DG2 activates FoxO similar to, but independently of, LRRK2. However, in spite of the similar activation mechanism, the genetic results suggested that the Hid-DIAP-Dronc pathway is not a major cause of the optic degeneration by DG2-FoxO (Fig. S8A–D). Supporting this result, a quantitative RT-PCR analysis showed that DG2 or DG2/dFoxO does not effectively stimulate FoxO-mediated transactivation of *hid* as well as *4E-BP* (Fig. S8E and F). We attempted to determine downstream effector(s) of DG2-dFoxO using a combination of microarrays, real-time PCR and *Drosophila* genetic screening, but could not identify any candidate genes, suggesting that DG2 has more complex functions in gene regulation. For example, DG2 might modulate another transcription regulator through phosphorylation along with dFoxO.

Activation of the NOS-sGC pathway leads to increased cGMP levels [26], which in turn has physiological consequences by regulating cGMP effector proteins such as cGMP-regulated ion channels, cGMP-regulated phosphodiesterases, and cGKs [25], [27]. It is widely appreciated that cGKs have a variety of roles in tissues, and in the central nervous system. For instance, cGKs regulate neurotransmitter release/uptake and receptor trafficking, neuronal differentiation and axon guidance, synaptic plasticity and memory through the phosphorylation of substrates [27], [28], [29]. There are two cGK isoforms, cGKI α/β and cGKII, in vertebrates. While cGKI α/β is cytosolic and mainly found in the cerebellum, cerebral cortex, hippocampus, hypothalamus, and olfactory bulb of the brain, cGKII is located in the cellular membranes and widely distributed in the brain [30], [31], [32]. Here, we demonstrated that cGKII is abundantly expressed in DA neurons in the substantia nigra of the murine midbrain, suggesting that cGKII has a pathogenic role similar to DG2.

What signal mediates stimulation of cGMP synthesis and subsequent cGKII activation in PD remains unclear. The activation of microglia is believed to be one of the pathological processes [33], [34], which might begin with the release of aggregated proteins such as oligomeric α-synuclein from neurons into the extracellular space [35]. Inflammation will be amplified by microglial activation and the release of proinflammatory cytokines and inducible NOS [5]. Similarly, dNOS, the only NOS orthologue in *Drosophila*, is involved in an immune response [36]. Thus, inducible NOS responding early to inflammation could be a trigger of the cGKII-FoxO-mediated neurotoxic pathway in humans. In this context, pathogenic LRRK2 with increased kinase activity might potentiate the above pathogenic mechanism. We found that cGKII physically interacts with LRRK2 (Fig. S9), and that they are co-localized at the endosomes (Fig. S10) although our current study suggests LRRK2 and cGKII act independently in the context of FoxO activation. However, we observed that co-expression of cGKII KD and LRRK2 3KD partially stimulates FoxO (Fig. 4B). These kinases have been reported to form a dimmer when activated [29], [37], [38]. Thus overexpression of kinase-dead forms of cGKII and LRRK2 may accidentally recruit and activate the endogenous kinases in 293T cells although we could not detect the endogenous expression of cGKII in this cell line.

The involvement of NO signaling in PD has been suggested by the findings of higher levels of nNOS and iNOS in the nigrostriatal region and basal ganglia in post mortem PD brains [3], [4]. The emerging evidence for pathogenic roles of microglia and astrocytes in PD now supports the idea that glia-induced inflammation and NO production promote the disease's development. However, most studies with post mortem samples or PD models showed only that NO could be a generator of oxidative stress since NO is a free radical involved in a wide range of physiologic events [39]. A very recent study on rodent models of PD have shown that specific inhibition of sGC-cGMP signaling improves basal ganglia dysfunction and motor symptoms, suggesting that NO signaling could act specifically on PD etiology [40]. Our study here provides the possibility that NO signaling downstream to cGK along with FoxO has a pathogenic role in PD.

The relationship between the NO signal and FoxO has been pointed out in a report on a tail suspension-induced model of muscle atrophy, where nNOS-NO is suggested to induce muscle atrophy by upregulating the muscle-specific E3 ubiquitin ligases MuRF-1 and atrogin-1/MAFbx through FoxO activation. Since, the AKT signal is not involved in this mechanism, the molecular mechanism by which FoxO is regulated by nNOS-NO remains unknown [41]. Considering our finding regarding neurodegeneration, cGK may regulate FoxO as a mediator of the NO signal in the atrophic muscles as well. Studies have shown that cGK indirectly activates FoxO4 through activation of the JNK pathway [42], [43], which provides anti-tumor effects in colon cancer cells. Although the proposed sites of phosphorylation by JNK do not appear to be conserved in dFoxO, there is substantial evidence that JNK-FoxO regulates different cellular processes including anti-aging and cell death in *Drosophila* [44], [45], [46]. Thus, DG2 could also stimulate the JNK pathway in conjunction with FoxO, widely affecting a variety of cellular mechanisms. This idea could explain why the FoxO SA mutant failed to suppress the DG2-mediated decrease in lifespan of *Drosophila* (Fig. 6E and F).

Although more studies are needed in mammalian systems, our finding of a novel link between the NO signal and FoxO in neurodegeneration suggests that appropriate pharmacological control of the NO pathway would prevent or diminish pathological problems in PD.

## Materials and Methods

### *Drosophila* genetics

The *Drosophila* cultures and crosses were performed on standard fly food containing yeast, cornmeal and molasses, and flies were raised at 25°C unless otherwise stated. General fly stocks and *GAL4* lines were obtained from the Bloomington *Drosophila* stock center. These flies have been described previously: *UAS-dFoxO* [47], *UAS-dFoxO S259A* [16], *UAS-DG1* [18], *UAS-DG2* [18], *UAS-dNOS* [48], *UAS-hLRRK2 WT* [49], *UAS-hLRRK2 I2020T* [49], *UAS-dLRRK WT* [14], *UAS-dLRRK I1915T* [14], *UAS-dLRRK 3KD* [14], *e03680* (*dLRRK null*) [14], *elav*-GeneSwitch [50], *UAS–hipo/MST* [51], *UAS-dIKKß* [52], *UAS-CKIα RNAi* [53], *dFoxO^21^* [54], *dNOS^Δ15^* [55], *UAS-AKT1* (Bloomington stock #8191), *UAS-CDK1-Myc* (#6642), *UAS-CDK2-Myc* (#6634), *UAS-bsk/JNK* (#6407), *mnb^EY14320^/DYRK1^EY14320^* (#21430), *CkIα^EP1555^* (#17009, [56]), *DG2^k04703^* (#10382), *UAS-sGCα99B^RNAi^* (#28748), *UAS-sGCβ100B^RNAi^* (#28786), *hid^1^* (#631), *DIAP^1^* (#618), and *UAS-Dronc ^RNAi^* (NIG-fly 8091R-2 III). *UAS-human cGKII* was generated in the Davies lab.

### Antibodies

The anti-α-Tubulin (DM1A), anti-β-Tubulin (Tub2.1) and anti-FLAG (M2) antibodies were purchased from Sigma-Aldrich. The anti-FoxO1 (#9454) antibody was obtained from Cell Signaling Technology. The anti-Myc (4A6), anti-Actin (MAB1501) and anti-phospho-FoxO1 (Ser319, 51136-1) antibodies were purchased from Millipore, Chemicon and Signalway, respectively. The rabbit anti-*Drosophila* TH and anti-dFoxO polyclonal antibody has been described previously [16], [57]. Anti-cGKII [30] and anti-cGKIα [31] were kindly provided by Drs. M. Hoffmeister and P. Weinmeister, respectively. The rabbit anti-hLRRK2 polyclonal antibodies were raised against GST-hLRRK2 (823–1004 aa) and (1868–2138 aa) produced in *E. coli* BL21(DE3)pLysS (Novagen).

### Plasmids

cDNA for human cGKIα and rat cGKII, kindly provided by Drs. S. Lohmann and A. Smolenski, was subcloned into pcDNA3-Myc or pcDNA3-FLAG. A plasmid for EGFP-FoxO1 was a kind gift from Dr. T. Unterman. A plasmid for AKT-PH-GFP was from Addgene. Plasmids for *FLAG-hLRRK2* and *FLAG-dLRRK* [14], mouse FoxO1, and human 4E-BP1 and the luciferase reporter plasmid for FoxO (TK.IRS3) have been reported elsewhere [58]. The plasmid for DG2 was also reported previously [18]. Mutations were introduced using the QuikChange II XL Site-directed mutagenesis kit (Stratagene). Although we used mouse FoxO1 cDNA as a mammalian FoxO gene, the numbering is based on the human sequence to avoid confusion. Thus, Ser149–152, Ser181 and Ser316 in mouse FoxO1 correspond to Ser152–155, Ser184 and Ser319 in human FoxO1, respectively. The kinase-dead form of rat cGKII (cGKII KD) was generated by replacing Asp549 with alanine, which corresponds to bovine cGKIα D501A mutation described in [59].

### *In vitro* phosphorylation assay

FLAG-cGKII, FLAG-hLRRK2, and mock fractions immunopurified from transfected and mock-transfected 293T cells were used as kinase sources. The same batches of kinase fractions were used throughout the experiments, and their quality and quantity was confirmed by western blot as shown in Fig. 5B and S6. Five micrograms of GST-FoxO1, mutant forms of GST-FoxO1 and His-4E-BP1 were incubated with the kinase sources in a kinase reaction buffer containing 20 mM HEPES (pH7.4), 15 mM MgCl_2_, 5 mM EGTA, 0.1% Triton X-100, 0.5 mM DTT, 1 mM β-glycerolphosphate, and 2.5 µCi [γ-^32^P]-ATP in the presence or absence of 30 µM cGMP for 30 min at 30°C. The reaction mixture was then suspended in SDS sample buffer and subjected to SDS-PAGE and autoradiography.

### Cell culture, immunopurification and western blotting

Transfection of human embryonic kidney 293T and *Drosophila* Schneider 2 (S2) cells, immunopurification from the transfected cell or mouse brain lysate, and western blotting were performed as described previously [16], [60], [61]. Flp-In T-REx-293 cell line harboring doxycycline-inducible EGFP-FoxO1 gene was generated according to the manufacturer's instructions (Invitrogen).

### Scanning Electron Microscopy (SEM)

Adult flies were processed as described previously [14]. SEM images were obtained at The Biomedical Research Core of Tohoku University Graduate School of Medicine.

### Lifespan and survival assays

Twenty female adult flies per vial were maintained at 29°C, transferred to fresh fly food vials containing 250 µl of yeast paste and 25 µg/ml of RU486, and scored for survival every 4 days. To control for isogeny, all fly lines were backcrossed to the *w^−^* wild-type background for six generations or were generated on the *w* ^−^ background, and thus have matched genetic backgrounds. Survival assays of flies treated with 2 mM paraquat were performed as described previously [14].

### Climbing assay

The climbing assay was performed as described previously [14]. Briefly, twenty flies were placed in a plastic vial (18.6 cm in height×3.5 cm^2^ in area) and gently tapped to bring them down to the bottom of the vial. Flies were given 18 s to climb and the number of flies more than 6 cm from the bottom was counted. Twenty trials were performed for the same set of flies. Flies at 20 days of age were left untreated or treated with 1 mM L-DOPA for 4 days, then subjected to climbing assays.

### Whole-mount immunostaining

Total number of TH-positive neurons were calculated following whole-mount immunostaining of brain samples as described previously [57]. All immunohistochemical analyses were performed using a Carl Zeiss laser scanning microscope system.

### Statistical analysis

The one-way repeated measures ANOVA was used to determine significant differences between multiple groups unless otherwise indicated. If a significant result was achieved (*p*<0.05), the means of the control and the specific test group were analyzed using the Tukey-Kramer test. For lifespan assays, a Kaplan-Meier analysis with the log-rank test was performed.

## Supporting Information

Figure S1 **Evaluation of** ***mnb*** **and** ***dg1*** **expression in** ***mnb^EY14320^*** **and** ***UAS-DG1*** **fly lines in the presence of the** ***GAL4*** **driver.** Total RNA was extracted from the *Da-Gal4* crosses. The *mnb*, an orthologue of mammalian *DYRK1*, *dg1* and *rp49* transcript levels were measured by real-time PCR. *mnb* (**A**) or *dg1* (**B**) transcript levels normalized to those of *rp49* are presented.
(TIF)

Figure S2 **DG1 does not exacerbate dFoxO-mediated eye degeneration.** Transgenic expression of DG2 alone did not produce eye degeneration, and DG1 had little effect on the eye phenotype caused by expression of dFoxO (when compared to Figure 2B). (**C**) The numbers of ommatidia per fly eye (from 5 flies) were quantified. *, *p*<0.05; N.S., non-significant. The genotypes are: *UAS-DG2*; *GMR-Gal4* (**A**), *GMR-Gal4, UAS-dFoxO; UAS-DG1* (**B**), *GMR-Gal4/UAS-EGFP* (EGFP), *GMR-Gal4, UAS-dFoxO/UAS-EGFP* (dFoxO, EGFP), *GMR-Gal4, UAS-dFoxO; UAS-DG1* (dFoxO, DG1) (**C**).
(TIF)

Figure S3 **cGKII does not form a stable complex with cGKIα or FoxO1.** Lysate from 293T cells transfected with cGKII-FLAG together with or without FoxO1-Myc or cGKIα-Myc was immunoprecipitated with anti-FLAG antibody (FLAG-IP). Immunoprecipitates and total soluble lysates (Lysate) were analyzed by western blotting.
(TIF)

Figure S4 **cGKII is expressed in DA neurons of the murine midbrain.** Immunolocalization of cGKIα (green in **A, C**), cGKII (green in **B, D**) and TH (red) in coronal sections of the substantia nigra (**A, B**) and striatum (**C, D**) of the brain. Yellow in **B** indicates the expression of cGKII in TH-positive neuronal processes (arrow heads) as well as cell bodies (arrows). The right columns of each panel show high-magnification images of the boxes in the left columns. Scale bars = 20 µm.
(TIF)

Figure S5 **Mutations of cGKII phosphorylation sites localized in FoxO1-N do not affect the FoxO-transcriptional activity.** (**A**) Reported phosphorylation sites in FoxO1 by cGKI are depicted [22]. Phospho-resistant mutants, where the indicated Ser or Thr residues are replaced with alanine, are also shown. (**B**) The phospho-signal by cGKII was decreased in GST-FoxO-4M compared with GST-FoxO-N WT (lane 3 *vs.* lane 2), but was no longer decreased in GST-FoxO-5M (data not shown), suggesting that S184 is not a major phosphorylation site by cGKII. (**C**) The FoxO1 4M mutation had little effect on FoxO-transcriptional activity stimulated by cGKII and/or LRRK2. (**D**) Effects of the 4M mutation on physical interaction between FoxO1 and 14-3-3ε were estimated in 293T cells. FoxO1-Myc-6x His was pulled down with Ni-NTA beads from the lysate of cells expressing the indicated transgenes.
(TIF)

Figure S6 **The Ser319 site of FoxO1 is not a major target of cGKI** ***in vitro*** **.** *In vitro* kinase assay was performed as in Fig. 5. P3 SA; a P3 mutant in which the Ser319 residue is replaced with alanine. Autophosphorylation signals of cGKII and cGKI are also shown in the upper panel.
(TIF)

Figure S7 **cGKII phosphorylates LRRK2.** (**A**) cGKII WT but not cGKII KD phosphorylates LRRK2 3KD (lane 9) as well as LRRK2 WT (lane 6) in *in vitro* kinase assay. *In vitro* kinase assay was performed as in Fig. 5. (**B**) Western blot analysis with anti-FLAG indicates similar amounts of FLAG-LRRK2 WT and FLAG-LRRK2 3KD were used in the kinase assay.
(TIF)

Figure S8 **Hid is not a major gene responsible for FoxO-DG2-mediated optic degeneration.** Introduction of loss-of-function alleles of a pro-apoptotic gene *hid* (**B**) or anti-apoptotic *DIAP* (**C**), or knockdown of Dronc, a caspase downstream of Hid (**D**), had little effects on the eye phenotype by co-expression of dFoxO and DG2 (**A**). The genotypes are: *UAS-DG2; GMR-Gal4, UAS-dFoxO* (**A**), *UAS-DG2; GMR-Gal4, UAS-dFoxO; hid^1^* (**B**), *UAS-DG2; GMR-Gal4, UAS-dFoxO; DIAP^1^* (**C**), *UAS-DG2; GMR-Gal4, UAS-dFoxO; UAS-Dronc^RNAi^* (**D**). (**E**) Real-time RT-PCR analysis for *hid* and *4E-BP* was performed using total RNA from S2 cells expressing the indicated gene combinations. Values are presented as the mean ± SE for three repeated experiments. *, *p*<0.05 *vs.* Control.
(TIF)

Figure S9 **cGKII is associated with LRRK2.** (**A**) Lysate from 293T cells transfected with FLAG-tagged LRRK2 with or without Myc-cGKII was immunoprecipitated with anti-FLAG antibody (FLAG-IP). Immunoprecipitates and total soluble lysates (lysate) were analyzed by western blotting. (**B**) The diagram represents LRRK2 and the mutants used to determine the cGKII-binding domain. Numbers in parentheses indicate corresponding amino acid residues of LRRK2. LRR, leucine-rich repeat; ROC, Ras in complex proteins; COR, C-terminal of Roc; Kinase, protein kinase domain; WD40, WD40 domain. (**C**) Immunoprecipitation-western blot analysis as in (**A**) revealed cGKII to be associated with LRRK2-C. (**D**) cGKII associates strongly with LRRK2-C_3_, and weakly with LRRK2–C_1_ and –C_2_. (**E**) Endogenous interaction of cGKII but not cGKIα with LRRK2 in brain tissue. Mouse brain tissues were lysed as described [60], then the supernatant fractions were immunoprecipitated (IP) with anti-cGKII or anti-cGKIα antibodies. The co-precipitated LRRK2 was detected by western blotting using anti-LRRK2 antibody. 293T lysate expressing FLAG-LRRK2 or FLAG-cGKII served as a positive control.
(TIF)

Figure S10 **cGKII is co-localized with LRRK2 at the endosomes.** (**A**) Immunolocalization of cGKII and LRRK2 in 293T cells expressing FLAG-LRRK2 and Myc-cGKII. cGKII and LRRK2 were visualized with anti-Myc (green) or anti-LRRK2 antibody (red). LRRK2 is localized at the Rab-positive endosomes (data not shown). cGKII is localized at the cytoplasmic membrane and partly in the cytoplasmic compartments. cGKII and LRRK2 were co-localized at the Rab-positive endosomes (yellow). Scale bar = 10 µm. (**B**–**E**) Immunolocalization of cGKII (red) in 293T cells expressing Myc-cGKII and EGFP-tagged Rabs (green). Cytosolic cGKII is located mainly at Rab4- and Rab5-positive endosomes, and partially at Rab7- or Rab11-positive endosomes.
(TIF)
