# Supplementary figures and images for: The Nitric Oxide-Cyclic GMP Pathway Regulates FoxO and Alters Dopaminergic Neuron Survival in Drosophila
Source: PLoS One. 2012 Feb 29;7(2):e30958. doi: 10.1371/journal.pone.0030958 (PMC3290610; doi:10.1371/journal.pone.0030958)

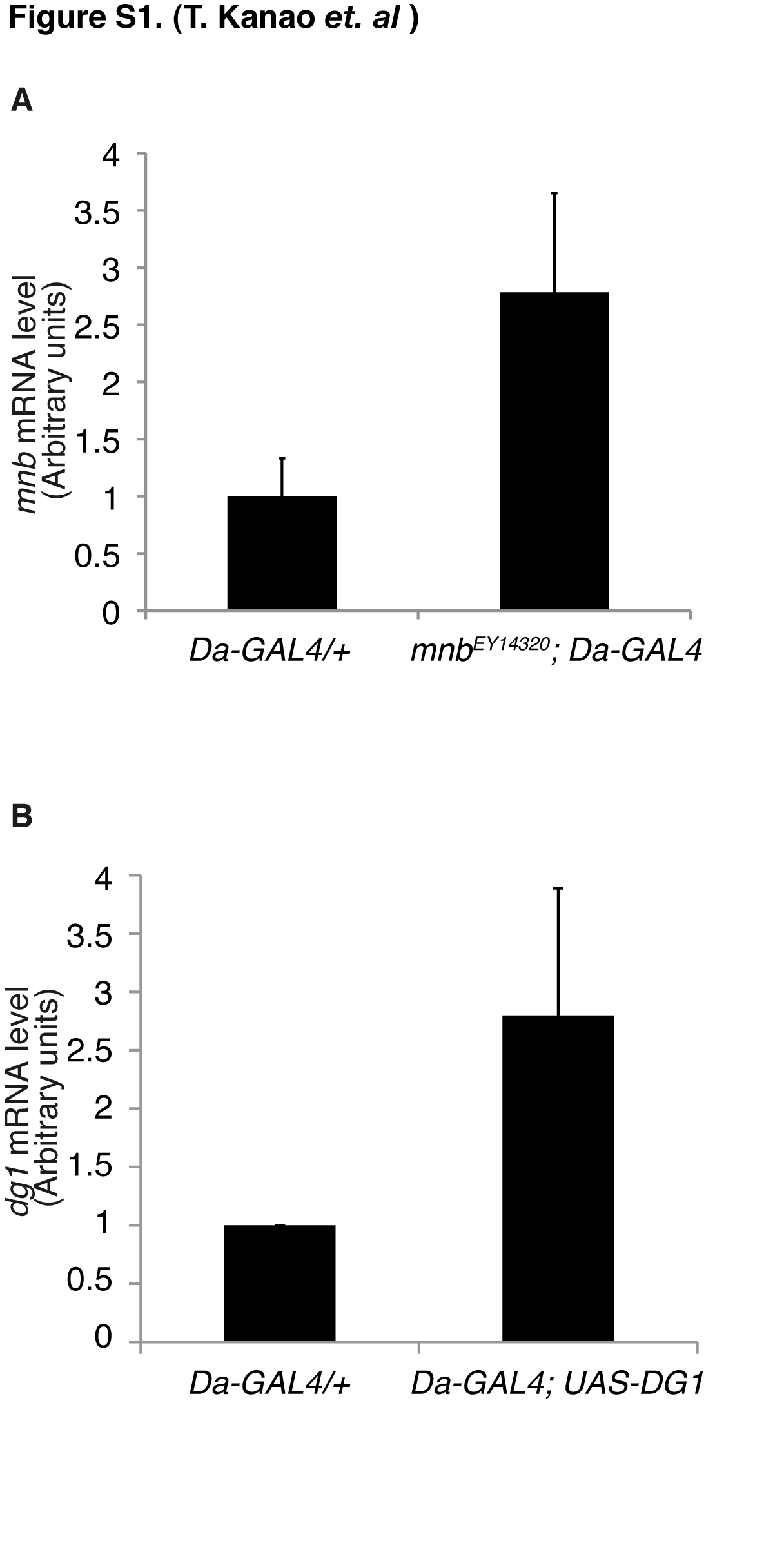

Supplement: Figure S1 — Evaluation of mnb and dg1 expression in mnbEY14320 and UAS-DG1 fly lines in the presence of the GAL4 driver. Total RNA was extracted from the Da-Gal4 crosses. The mnb, an orthologue of mammalian DYRK1, dg1 and rp49 transcript levels were measured by real-time PCR. mnb (A) or dg1 (B) transcript levels normalized to those of rp49 are presented. (TIF) [file pone.0030958.s001.tif]

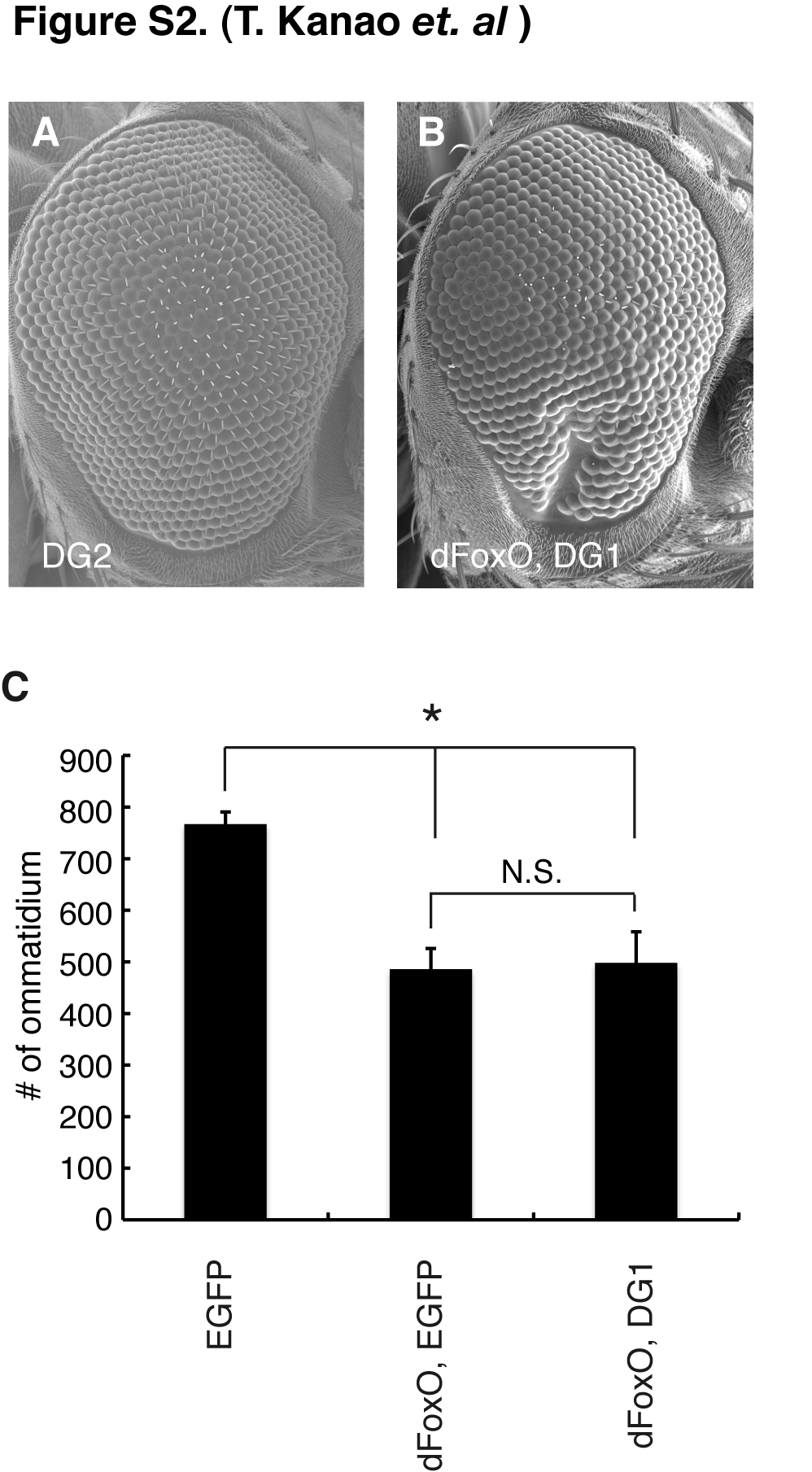

Supplement: Figure S2 — DG1 does not exacerbate dFoxO-mediated eye degeneration. Transgenic expression of DG2 alone did not produce eye degeneration, and DG1 had little effect on the eye phenotype caused by expression of dFoxO (when compared to Figure 2B). (C) The numbers of ommatidia per fly eye (from 5 flies) were quantified. *, p<0.05; N.S., non-significant. The genotypes are: UAS-DG2; GMR-Gal4 (A), GMR-Gal4, UAS-dFoxO; UAS-DG1 (B), GMR-Gal4/UAS-EGFP (EGFP), GMR-Gal4, UAS-dFoxO/UAS-EGFP (dFoxO, EGFP), GMR-Gal4, UAS-dFoxO; UAS-DG1 (dFoxO, DG1) (C). (TIF) [file pone.0030958.s002.tif]

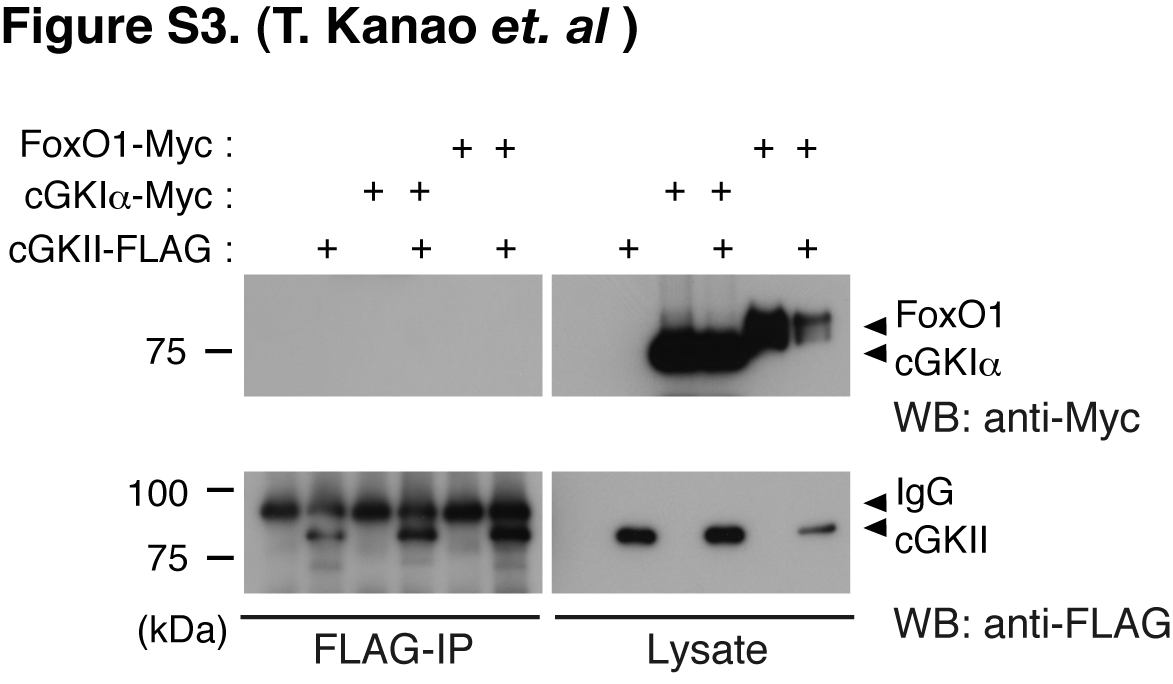

Supplement: Figure S3 — cGKII does not form a stable complex with cGKIα or FoxO1. Lysate from 293T cells transfected with cGKII-FLAG together with or without FoxO1-Myc or cGKIα-Myc was immunoprecipitated with anti-FLAG antibody (FLAG-IP). Immunoprecipitates and total soluble lysates (Lysate) were analyzed by western blotting. (TIF) [file pone.0030958.s003.tif]

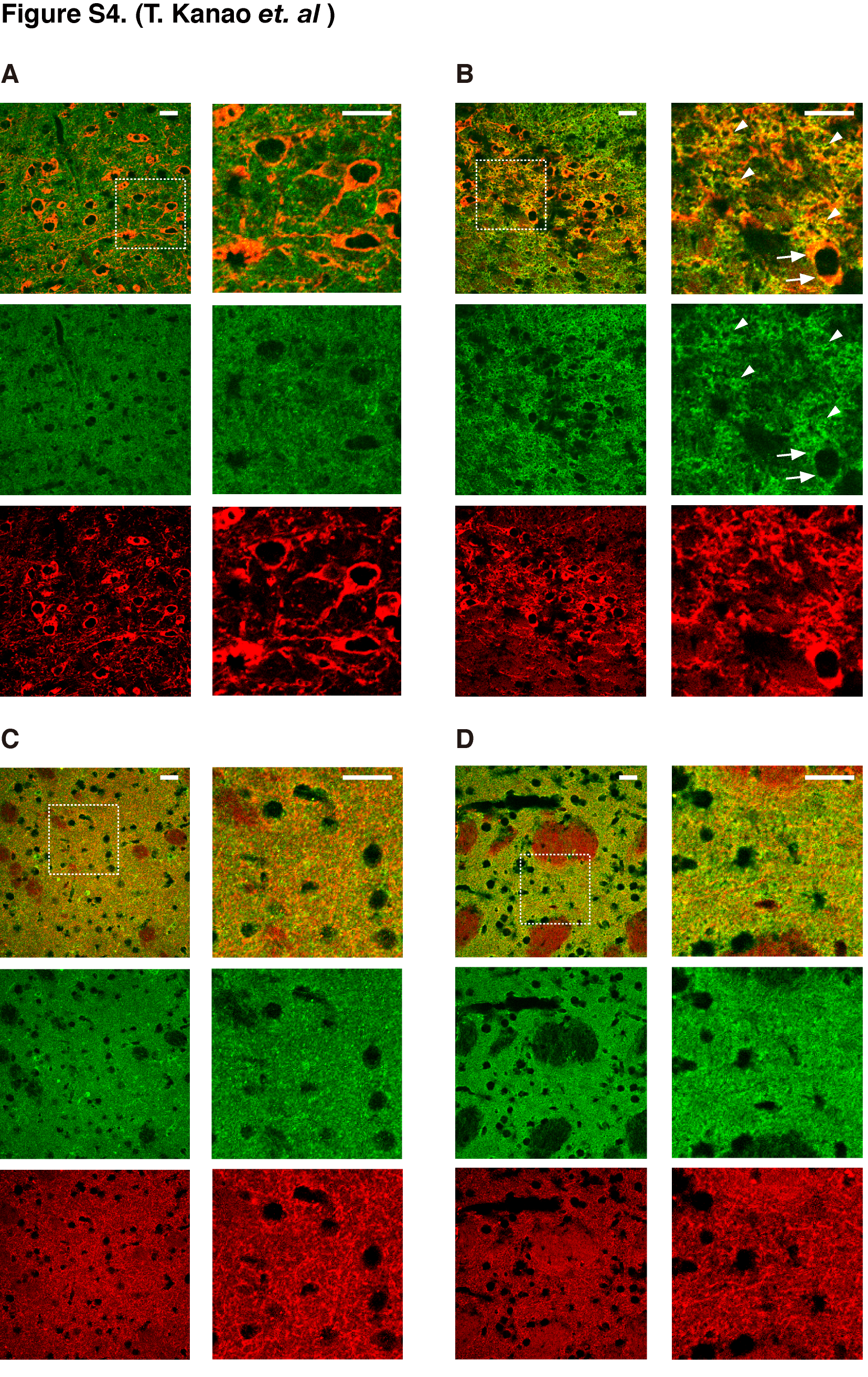

Supplement: Figure S4 — cGKII is expressed in DA neurons of the murine midbrain. Immunolocalization of cGKIα (green in A, C), cGKII (green in B, D) and TH (red) in coronal sections of the substantia nigra (A, B) and striatum (C, D) of the brain. Yellow in B indicates the expression of cGKII in TH-positive neuronal processes (arrow heads) as well as cell bodies (arrows). The right columns of each panel show high-magnification images of the boxes in the left columns. Scale bars = 20 µm. (TIF) [file pone.0030958.s004.tif]

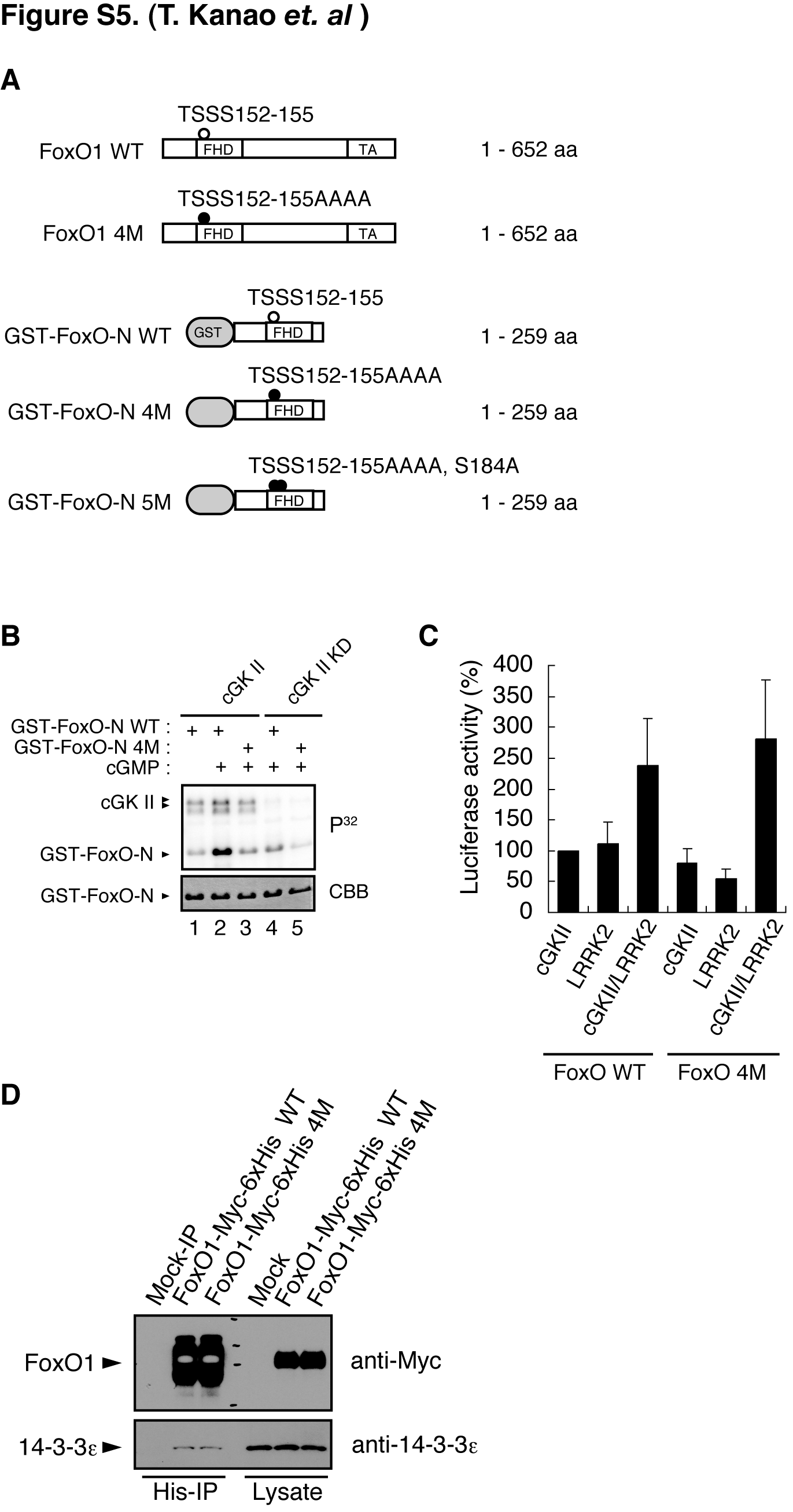

Supplement: Figure S5 — Mutations of cGKII phosphorylation sites localized in FoxO1-N do not affect the FoxO-transcriptional activity. (A) Reported phosphorylation sites in FoxO1 by cGKI are depicted [22]. Phospho-resistant mutants, where the indicated Ser or Thr residues are replaced with alanine, are also shown. (B) The phospho-signal by cGKII was decreased in GST-FoxO-4M compared with GST-FoxO-N WT (lane 3 vs. lane 2), but was no longer decreased in GST-FoxO-5M (data not shown), suggesting that S184 is not a major phosphorylation site by cGKII. (C) The FoxO1 4M mutation had little effect on FoxO-transcriptional activity stimulated by cGKII and/or LRRK2. (D) Effects of the 4M mutation on physical interaction between FoxO1 and 14-3-3ε were estimated in 293T cells. FoxO1-Myc-6x His was pulled down with Ni-NTA beads from the lysate of cells expressing the indicated transgenes. (TIF) [file pone.0030958.s005.tif]

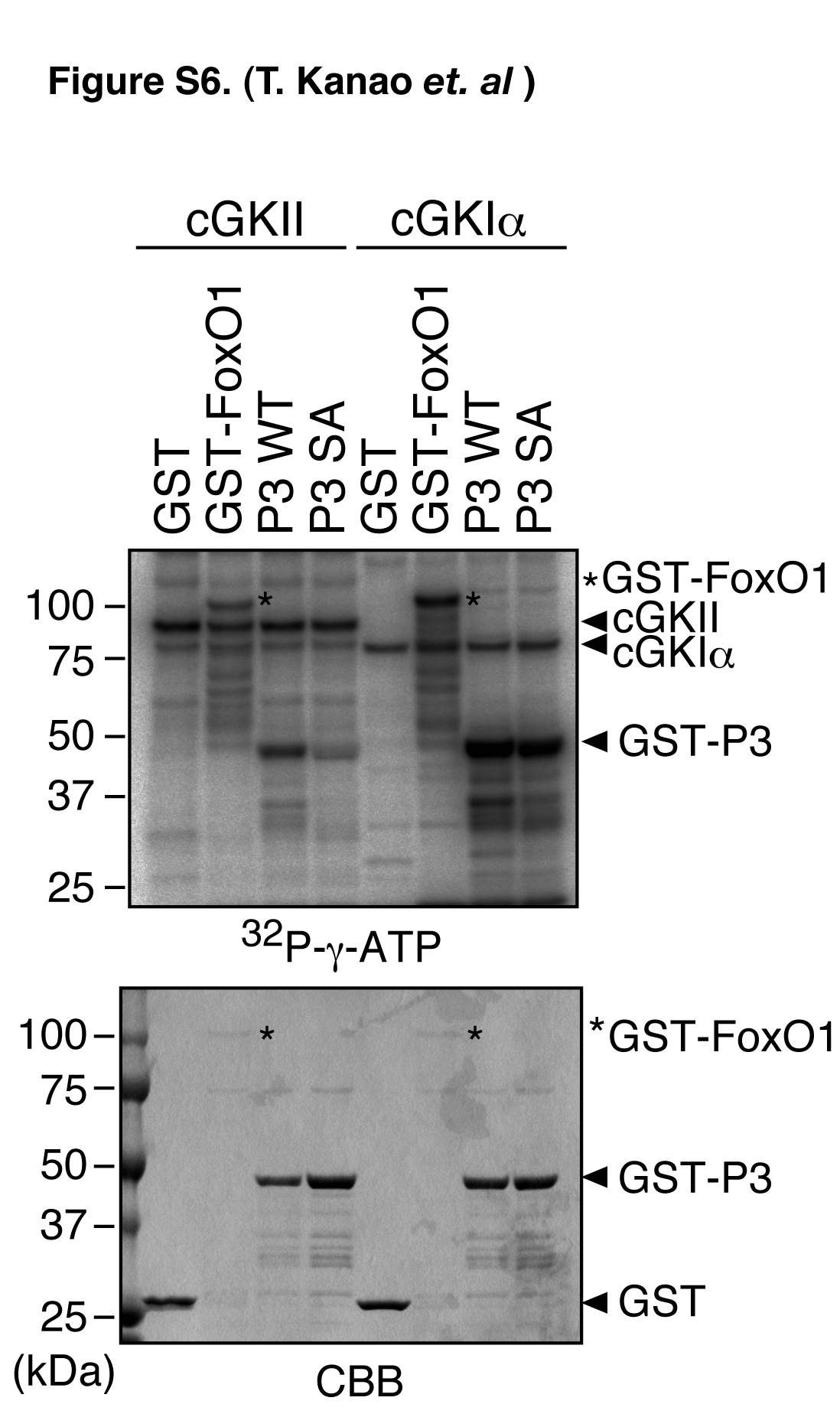

Supplement: Figure S6 — The Ser319 site of FoxO1 is not a major target of cGKI in vitro . In vitro kinase assay was performed as in Fig. 5. P3 SA; a P3 mutant in which the Ser319 residue is replaced with alanine. Autophosphorylation signals of cGKII and cGKI are also shown in the upper panel. (TIF) [file pone.0030958.s006.tif]

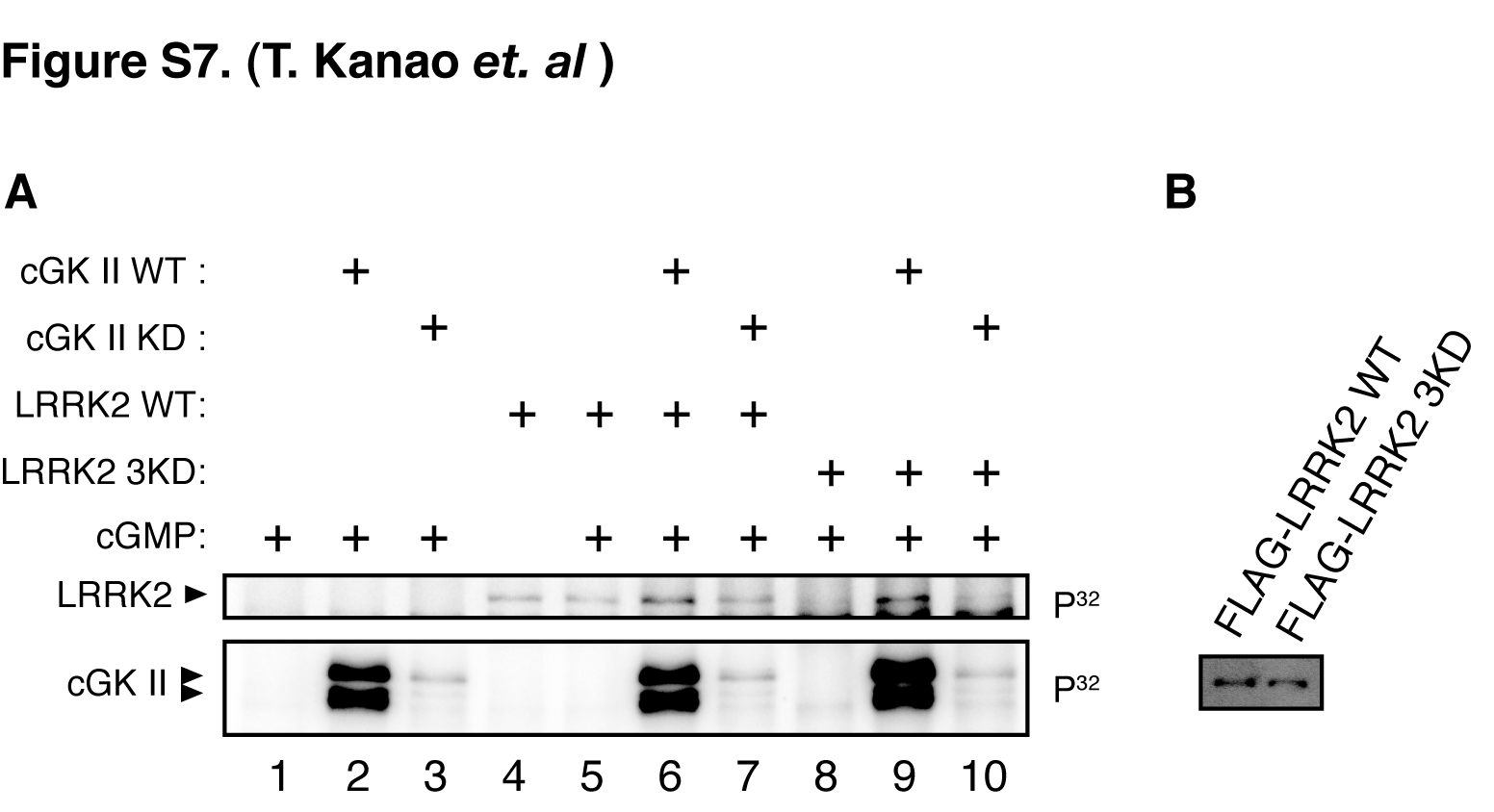

Supplement: Figure S7 — cGKII phosphorylates LRRK2. (A) cGKII WT but not cGKII KD phosphorylates LRRK2 3KD (lane 9) as well as LRRK2 WT (lane 6) in in vitro kinase assay. In vitro kinase assay was performed as in Fig. 5. (B) Western blot analysis with anti-FLAG indicates similar amounts of FLAG-LRRK2 WT and FLAG-LRRK2 3KD were used in the kinase assay. (TIF) [file pone.0030958.s007.tif]

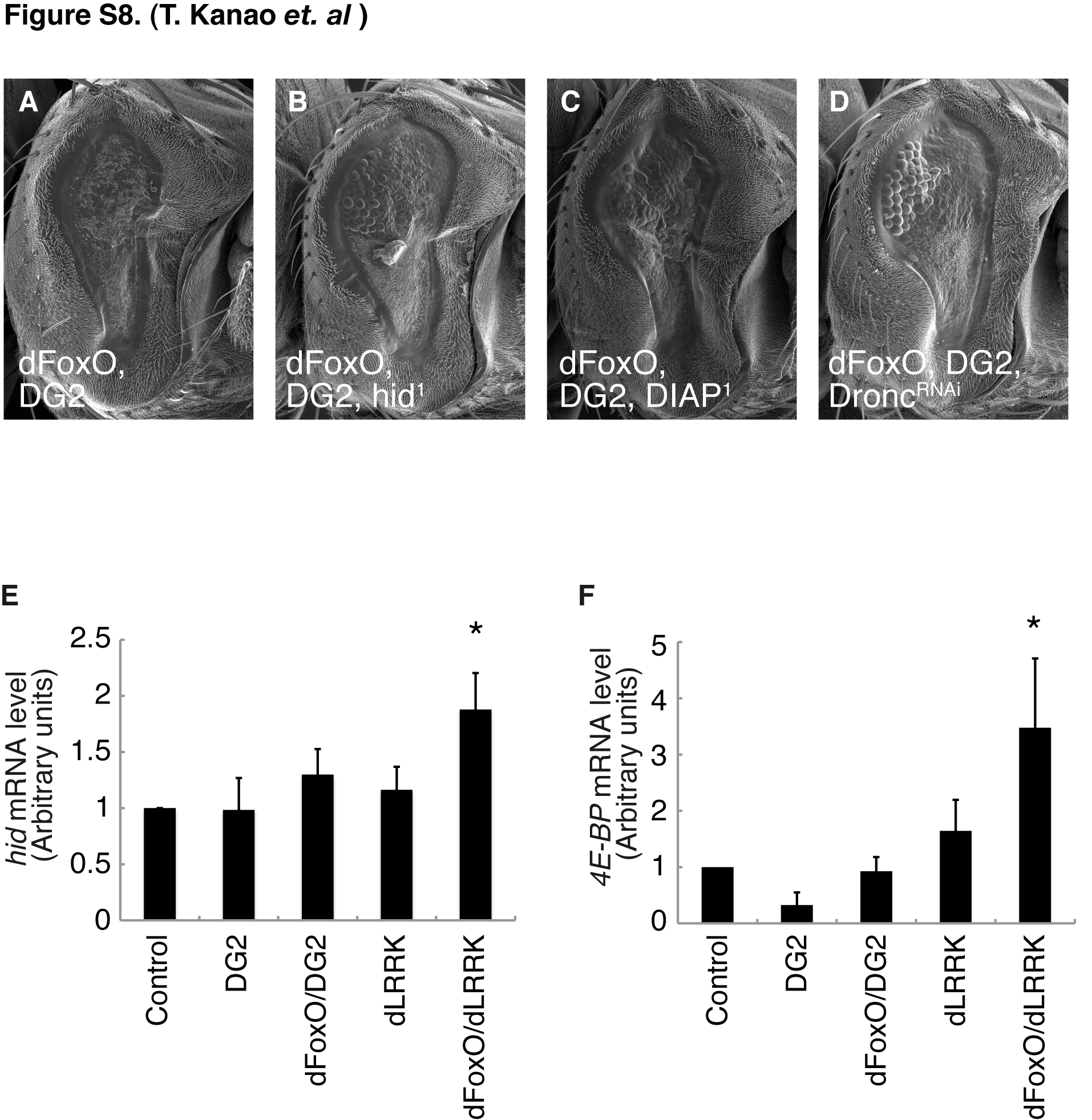

Supplement: Figure S8 — Hid is not a major gene responsible for FoxO-DG2-mediated optic degeneration. Introduction of loss-of-function alleles of a pro-apoptotic gene hid (B) or anti-apoptotic DIAP (C), or knockdown of Dronc, a caspase downstream of Hid (D), had little effects on the eye phenotype by co-expression of dFoxO and DG2 (A). The genotypes are: UAS-DG2; GMR-Gal4, UAS-dFoxO (A), UAS-DG2; GMR-Gal4, UAS-dFoxO; hid1 (B), UAS-DG2; GMR-Gal4, UAS-dFoxO; DIAP1 (C), UAS-DG2; GMR-Gal4, UAS-dFoxO; UAS-DroncRNAi (D). (E) Real-time RT-PCR analysis for hid and 4E-BP was performed using total RNA from S2 cells expressing the indicated gene combinations. Values are presented as the mean ± SE for three repeated experiments. *, p<0.05 vs. Control. (TIF) [file pone.0030958.s008.tif]

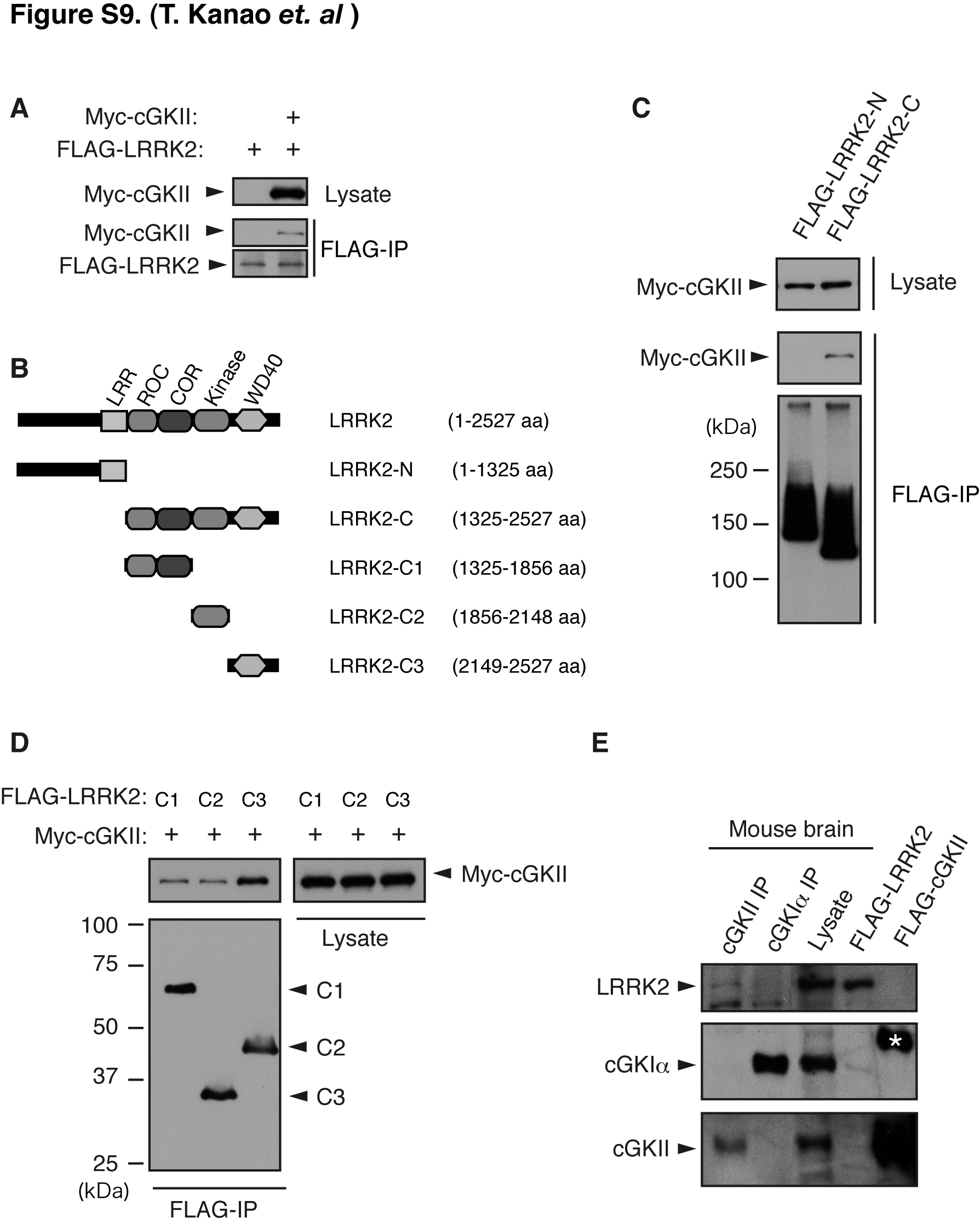

Supplement: Figure S9 — cGKII is associated with LRRK2. (A) Lysate from 293T cells transfected with FLAG-tagged LRRK2 with or without Myc-cGKII was immunoprecipitated with anti-FLAG antibody (FLAG-IP). Immunoprecipitates and total soluble lysates (lysate) were analyzed by western blotting. (B) The diagram represents LRRK2 and the mutants used to determine the cGKII-binding domain. Numbers in parentheses indicate corresponding amino acid residues of LRRK2. LRR, leucine-rich repeat; ROC, Ras in complex proteins; COR, C-terminal of Roc; Kinase, protein kinase domain; WD40, WD40 domain. (C) Immunoprecipitation-western blot analysis as in (A) revealed cGKII to be associated with LRRK2-C. (D) cGKII associates strongly with LRRK2-C3, and weakly with LRRK2–C1 and –C2. (E) Endogenous interaction of cGKII but not cGKIα with LRRK2 in brain tissue. Mouse brain tissues were lysed as described [60], then the supernatant fractions were immunoprecipitated (IP) with anti-cGKII or anti-cGKIα antibodies. The co-precipitated LRRK2 was detected by western blotting using anti-LRRK2 antibody. 293T lysate expressing FLAG-LRRK2 or FLAG-cGKII served as a positive control. (TIF) [file pone.0030958.s009.tif]

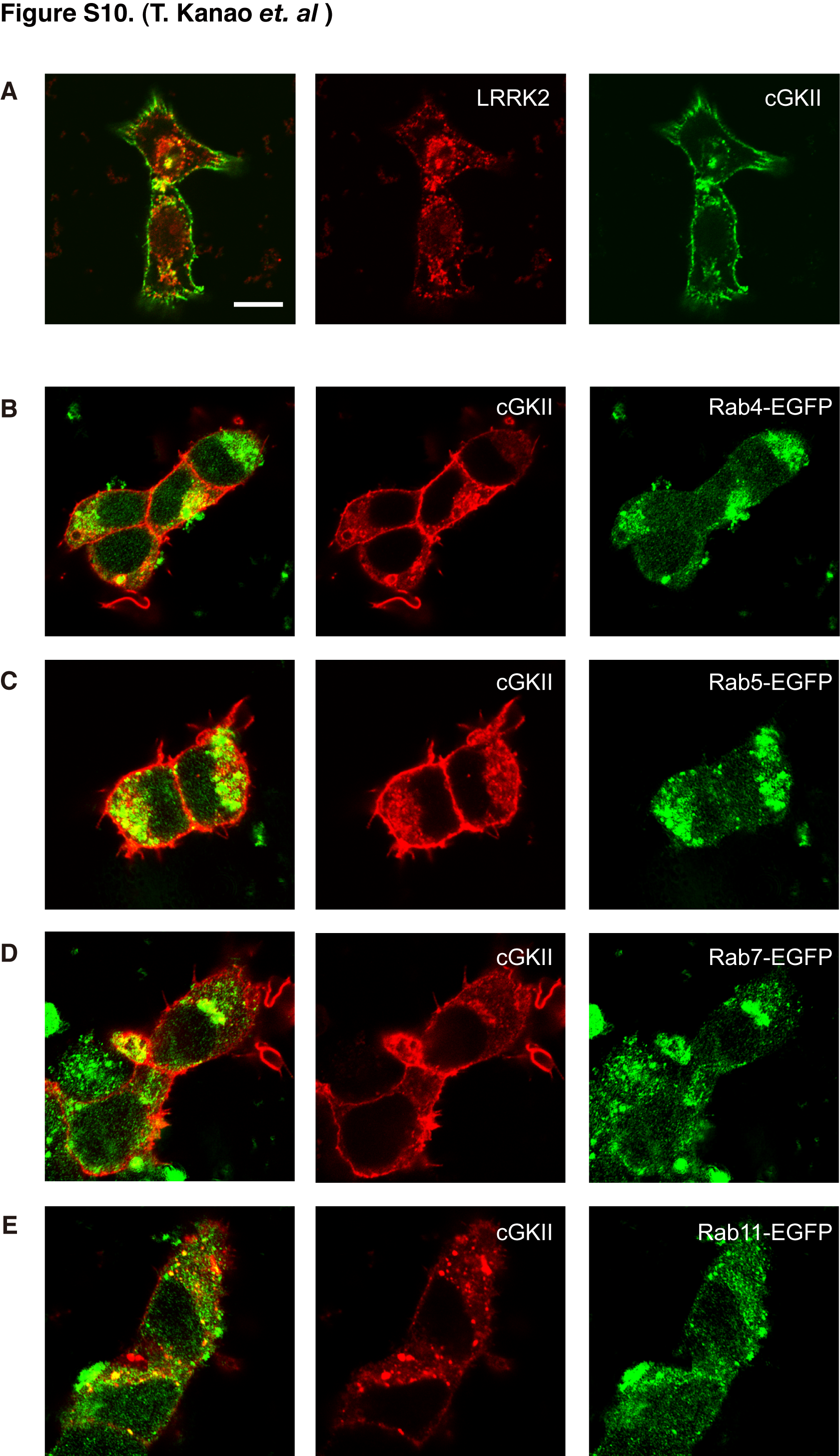

Supplement: Figure S10 — cGKII is co-localized with LRRK2 at the endosomes. (A) Immunolocalization of cGKII and LRRK2 in 293T cells expressing FLAG-LRRK2 and Myc-cGKII. cGKII and LRRK2 were visualized with anti-Myc (green) or anti-LRRK2 antibody (red). LRRK2 is localized at the Rab-positive endosomes (data not shown). cGKII is localized at the cytoplasmic membrane and partly in the cytoplasmic compartments. cGKII and LRRK2 were co-localized at the Rab-positive endosomes (yellow). Scale bar = 10 µm. (B–E) Immunolocalization of cGKII (red) in 293T cells expressing Myc-cGKII and EGFP-tagged Rabs (green). Cytosolic cGKII is located mainly at Rab4- and Rab5-positive endosomes, and partially at Rab7- or Rab11-positive endosomes. (TIF) [file pone.0030958.s010.tif]
